# KNTC1 initiates a KNTC1/E2F8/MYC positive feedback loop to facilitate tumorigenesis and enhance chemoresistance in bladder cancer

**DOI:** 10.1186/s13046-026-03651-4

**Published:** 2026-02-04

**Authors:** Kailai Chen, Hecheng Su, Feng Pei, Xi Chen, Meiqi Xu, Fang Chai, Yakun Luo

**Affiliations:** 1 NHC Key Laboratory of Molecular Probes and Targeted Diagnosis and Therapy, The Fourth Affiliated Hospital of Harbin Medical University, Harbin Medical University, Harbin, 150001 China; 2 Clinical Medical College, The Fourth Affiliated Hospital of Harbin Medical University, Harbin, 150001 China; 3 Key Laboratory for Photochemical Biomaterials and Energy Storage Materials of Heilongjiang Province, Key Laboratory for Photonic and Electronic Bandgap Materials of Ministry of Education, College of Chemistry and Chemical Engineering, Harbin Normal University, Harbin, 150025 China

**Keywords:** KNTC1, MYC, E2F8, Bladder cancer, PI3K/AKT/mTOR, Gemcitabine resistance

## Abstract

**Background:**

Bladder cancer (BLCA) is a lethal malignancy frequently challenged by chemoresistance and limited therapeutic options. Kinetochore-associated 1 (KNTC1) has been implicated in cancer, yet its precise role, regulatory mechanism, and therapeutic potential in BLCA remain largely unexplored.

**Methods:**

We analyzed KNTC1 expression and its clinical relevance using public databases (TCGA, GEO) and clinical specimens. Functional roles of KNTC1 in proliferation, invasion, metastasis, and gemcitabine resistance were assessed through in vitro and in vivo experiments (CCK-8, transwell, xenograft models). Mechanistic insights were gained via RNA-seq, co-immunoprecipitation, chromatin immunoprecipitation (ChIP), and luciferase reporter assays. A poly (ß-amino ester) (PAE)-based nanoparticle was synthesized for targeted in vivo delivery of KNTC1 siRNA.

**Results:**

KNTC1 was significantly upregulated in BLCA tissues, and its high expression correlated with poor patient prognosis. Functionally, KNTC1 promoted BLCA cell proliferation, invasion, metastasis, and gemcitabine resistance. Mechanistically, KNTC1 bound to the transcription factor E2F8 and facilitated its nuclear translocation, thereby enhancing E2F8-mediated transcriptional activation of MYC. MYC, in turn, transcriptionally upregulated KNTC1, forming a positive feedback loop that drove hyperactivation of the oncogenic PI3K/AKT/mTOR pathway. Silencing KNTC1, especially in combination with a MYC inhibitor (10058-F4), overcame gemcitabine resistance in vitro and in vivo. Therapeutic delivery of KNTC1 siRNA via a novel PAE nanocarrier (PAE@shKNTC1) significantly suppressed lung metastasis in a preclinical model.

**Conclusion:**

Our study identifies a novel KNTC1/E2F8/MYC positive feedback axis that drives BLCA tumorigenesis and chemoresistance. KNTC1 serves as both a prognostic biomarker and a promising therapeutic target, with targeted inhibition offering a potential novel strategy for BLCA treatment.

**Supplementary Information:**

The online version contains supplementary material available at 10.1186/s13046-026-03651-4.

## Background

Bladder cancer (BLCA), predominantly urothelial carcinoma, remains a significant global health burden, ranking among the most common malignancies worldwide [1]. It is characterized by high recurrence rates, substantial morbidity, and considerable mortality [2, 3]. The clinical management of BLCA presents formidable challenges, particularly in cases of muscle-invasive and metastatic disease [2]. While patients with non-muscle-invasive bladder cancer (NMIBC) often respond favorably to transurethral resection followed by intravesical therapy, those diagnosed with muscle-invasive bladder cancer (MIBC) or metastatic disease face a dramatically worse prognosis [4]. The standard first-line treatment for advanced BLCA continues to be platinum-based combination chemotherapy [5, 6]. However, therapeutic success is frequently compromised by the development of both intrinsic and acquired chemoresistance, leading to treatment failure and disease progression [7, 8]. Despite recent advances incorporating immune checkpoint inhibitors and targeted therapies into the treatment landscape [9], the overall survival benefits for patients with advanced BLCA remain modest, highlighting the persistent and urgent need for novel therapeutic approaches and more effective prognostic biomarkers.The complexity of BLCA pathogenesis and treatment resistance mechanisms underscores the necessity to identify and characterize molecular drivers that contribute to disease progression and therapeutic failure. The pursuit of novel molecular targets has become a central focus in BLCA research, with particular interest in proteins that regulate fundamental cellular processes that are frequently dysregulated in cancer [10].

Chromosomal instability (CIN), which refers to persistent errors in chromosome segregation during mitosis, is a well-established hallmark of cancer and contributes significantly to tumor evolution and heterogeneity [11]. In bladder cancer, CIN frequently constitutes a key feature of the genomic landscape and occurs more often in aggressive subtypes [12]. The kinetochore, a multiprotein complex that forms on centromeric regions, plays a central role in orchestrating chromosome segregation [13]. It provides not only the structural foundation for microtubule attachment but also actively monitors these attachments to ensure accurate chromosome distribution [14].

Kinetochore-associated protein 1 (KNTC1, also known as Rod, ZWILCH or ZW10), an evolutionarily conserved component of kinetochore complex, is indispensable for proper spindle assembly and chromosome segregation [15]. As a critical element of the mitotic checkpoint, KNTC1 helps safeguard the fidelity of chromosome segregation during cell division [16]. Disruption of KNTC1 function can lead to CIN, which is considered a pivotal event in tumorigenesis. Mounting evidence indicates that KNTC1 is upregulated in multiple tumor types, including non-small cell lung cancer (NSCLC) [17], hepatocellular carcinoma (HCC) [18], and esophageal squamous cell carcinoma (ESCC) [19]. Additionally, Huang et al. reported that KNTC1 may play a crucial role in the development of BLCA, however, the expression profile, clinical relevance, biological functions, and mechanistic contributions of KNTC1 in BLCA have not been comprehensively investigated [20].

More intriguingly, in this study, we integrated four publicly available transcriptomic datasets (GSE231383, GSE37815, GSE133624, and GSE188715) with our paired RNA sequencing data from normal human ureteral epithelial cells (SV-HUC-1) and bladder cancer cells (UMUC3) (SRA: PRJNA1394524). This integrated analysis identified four significantly upregulated genes in BLCA progression: KNTC1, ANLN, CCNB1, and HMMR. A comprehensive literature review revealed that, except for KNTC1, the biological functions and regulatory mechanisms of the other three genes [21–24] in bladder cancer have been relatively well studied. Therefore, we comprehensively studied the critical oncogenic role of KNTC1 in BLCA pathogenesis, progression, and chemoresistance, uncovering a novel regulatory axis and its therapeutic potential. We first demonstrated that KNTC1 is significantly overexpressed in BLCA tissues at both mRNA and protein levels, and its high expression strongly correlates with poor patient prognosis. Functional experiments revealed that KNTC1 knockdown inhibited, while its overexpression promoted, BLCA cell proliferation, migration, invasion, and cell cycle progression in vitro and in vivo. Mechanistically, RNA-seq and subsequent investigations identified that KNTC1 interacts with the transcription factor E2F8, promoting its nuclear translocation. This facilitates E2F8 binding to the *MYC* promoter, activating its transcription. The upregulated MYC further transcriptionally activates KNTC1, forming a positive feedback loop, and jointly drives the hyperactivation of the PI3K/AKT/mTOR signaling pathway. Furthermore, we discovered KNTC1 as a key driver of gemcitabine resistance, as its knockdown sensitized resistant cells to chemotherapy. Importantly, the therapeutic efficacy of targeting KNTC1 was validated using a novel targeted nanoparticle (PAEs-siKNTC1) delivery system, which significantly suppressed BLCA lung metastasis in a mouse model. Our findings position KNTC1 not only as a valuable prognostic biomarker but also as a promising therapeutic target for BLCA, operating through the novel KNTC1/E2F8/MYC signaling axis.

## Methods

### Cell culture

We purchased human uroepithelial cells SV-HUC-1 and human BLCA cell lines UMUC3, HT1197, SW780, J82, T24 and 5637 at the American Type Culture Collection. Culture media specifications were standardized as follows: 5637 in RPMI-1640; SW780 in L-15 Medium; SV-HUC-1 in F-12 K Medium; UMUC3/HT1197/J82 lineages in Eagle’s Minimum Essential Medium; T24 populations in McCoy’s 5a (all media reagents were sourced from Invitrogen, USA). Each medium was supplemented with 10% fetal bovine serum (FBS) and 1% penicillin-streptomycin. Cellular propagation was executed under controlled atmospheric conditions (37 °C, 5% CO2, humidity saturation). Cells were screened for *Mycoplasma* contamination biweekly using polymerase chain reaction assays.

### Cell counting kit-8 (CCK-8)

A total of 2 × 10³ cells were cultured in 96-well plates at a density of 2 × 10³cells per welll. Following experimental interventions, cellular responses were evaluated at specified intervals (0 h, 24 h, 48 h, 72 h, and 96 h post-treatment), Cell Counting Kit-8 solution (Abcam Biotech Co., Ltd.) was introduced to the wells. Absorbance values at 450 nm wavelength was subsequently measured using a microplate reader to assess cell viability.

#### Transwell assay

Matrigel-coated 24-well chambers were filled with medium containing 10% FBS, while cells resuspended in a serum-free medium were added to the upper chamber. The cells migrated through the filter attracted by the lower chamber medium. After 24 h, non-migratory cells were eliminated, and the migrating cells were fixed with methanol and stained with crystal violet before being eimaged under an inverted fluorescence microscope (Olympus IX73).

### Colony-formation assay

Post-treatment cellular clonogenicity was evaluated by seeding 1 × 10³ cells/well in 6-well culture plates under standard incubation conditions (37 °C, 5% CO2). Following 14 days of continuous culture, colonies were methanol-fixed and stained with 0.1% crystal violet for macroscopic visualization. After air-drying, colony enumeration was performed, with triplicate independent experiments conducted for statistical validation.

### Wound healing assay

Cells were seeded into 6-well plates. Upon reaching 100% confluency, scratch wounds were generated in the cell monolayer using a sterile 200 µL pipette tip. Images of the wound areas were captured at 0 h and 24 h post-scratching using an inverted microscope at 100× magnification. The wound area was subsequently quantified using Image J software.

### RNA sequencing

RNA integrity was verified using NanoDrop 2000 spectrophotometry (Thermo Fisher) prior to polyA-selected mRNA enrichment. Sequencing library construction utilized the Hieff NGS™ MaxUp Dual-mode mRNA Library Prep Kit (YEASEN, 12301ES96) following manufacturer specifications, including unique barcode incorporation for sample multiplexing. Size selection (150–200 bp inserts) was achieved through magnetic bead-based purification (YEASEN, 12601ES56). Library amplification employed 2×Super CanaceTM High-Fidelity Mix with adapter-specific primers, followed by Qubit^®^ 2.0 Fluorometer quantification. Pooled libraries underwent paired-end sequencing on Illumina NovaSeq 6000 platforms.

### Immunohistochemistry (IHC)

Histologic sections from tissues embedded in paraffin wax underwent antigen retrieval in citrate buffer for 15 min. Subsequently, The sections were treated with normal goat serum for 30 min to prevent non-specific binding. The tissue sections were treated with primary antibody against: anti-KNTC1 (ab85996, Abcam), Ki67 (ab15580, Abcam) and incubated entire night at 4 °C. Subsequently, sections were incubated for 2 h with secondary antibodies (ab205719/ab205718; Abcam; 1:3000). Avidin-biotin peroxidase detection systems with DAB substrate were utilized to label the locations of antigens, subsequently counter staining with hematoxylin (ab220365, Abcam) was performed. Immunohistochemical signal intensity and positively stained areas of tissue sections were independently evaluated and scored by observers.

### Dual-luciferase reporter assay

RiboBio (Guangzhou) generated luciferase reporter vectors carrying wild-type binding sequences for KNTC1-MYC interaction and corresponding MYC mutants (construction strategies detailed in Supplementary materials and methods). These vectors were co-transfected with KNTC1 (RiboBio) into BLCA cells. Luciferase activity was assessed 48 h post-transfection using Promega’s Dual-Glo^®^ Luciferase Assay System (E2920) on a GloMax 96 instrument, following the manufacturer’s protocol.

### Mouse xenografts and treatments

BALB/c nude mice (male, 6-week-old) were procured from Vital River Laboratory (Beijing) and kept in rigorously controlled SPF accommodations. Subcutaneous xenograft models were established by injecting 4 × 106 UMUC3 cells into the flank region of each mouse. Tumor dimensions (length/width) were measured weekly, with volumetric calculations derived from the standard formula: (length × width²)/2. Following a 40 days observation period, mice were humanely sacrificed for tumor excision and mass determination. In lung metastasis models, 1 × 10^6^ cells were injected through tail veins. After 32 days, IVIS@ Lumina system (CLS, USA) was used to analyze the tumor metastasis after mice intraperitoneal injected with D-luciferin. Next, mice were sacrificed after IVIS measurement, and tumor tissues were excised for further detection. Harvested tissues underwent hematoxylin and eosin (H&E) staining and immunohistochemical (IHC) analysis according to established protocols. Prior to imaging, 150 mg/kg luciferin substrate (MCE) was intraperitoneally administered following manufacturer specifications.

### Bioinformatic analysis

Enrichment analysis was conducted via the Bioconductor’s cluster profile package (version 4.14.4) using R (version 4.3.3) to explore biological functions in KNTC1-konckdown (sh-KNTC1) and control (sh-Ctrl) BLCA cells. The heatmap of the gene was drawn by using the R (version 4.3.3) software “heatmap package”. The heatmap were demonstrated 4578 significant differential expression genes (DEGs) between sh-Ctrl BLCA cells and sh-KNTC1 BLCA cells. Differential expressed genes were identified using DESeq2 package with *P* value adjusted to lower than 0.05 and log2 fold change greater than 1.

### Statistical analysis

Triplicate biological replicates were systematically executed for all experimental protocols. Statistical comparisons employed Student’s t-test or two-way ANOVA (depending on data distribution) using GraphPad Prism 9.0, with *P* < 0.05 considered significant.

## Results

### KNTC1 upregulation in BLCA associates with poor patient prognosis

**Fig. 1:**
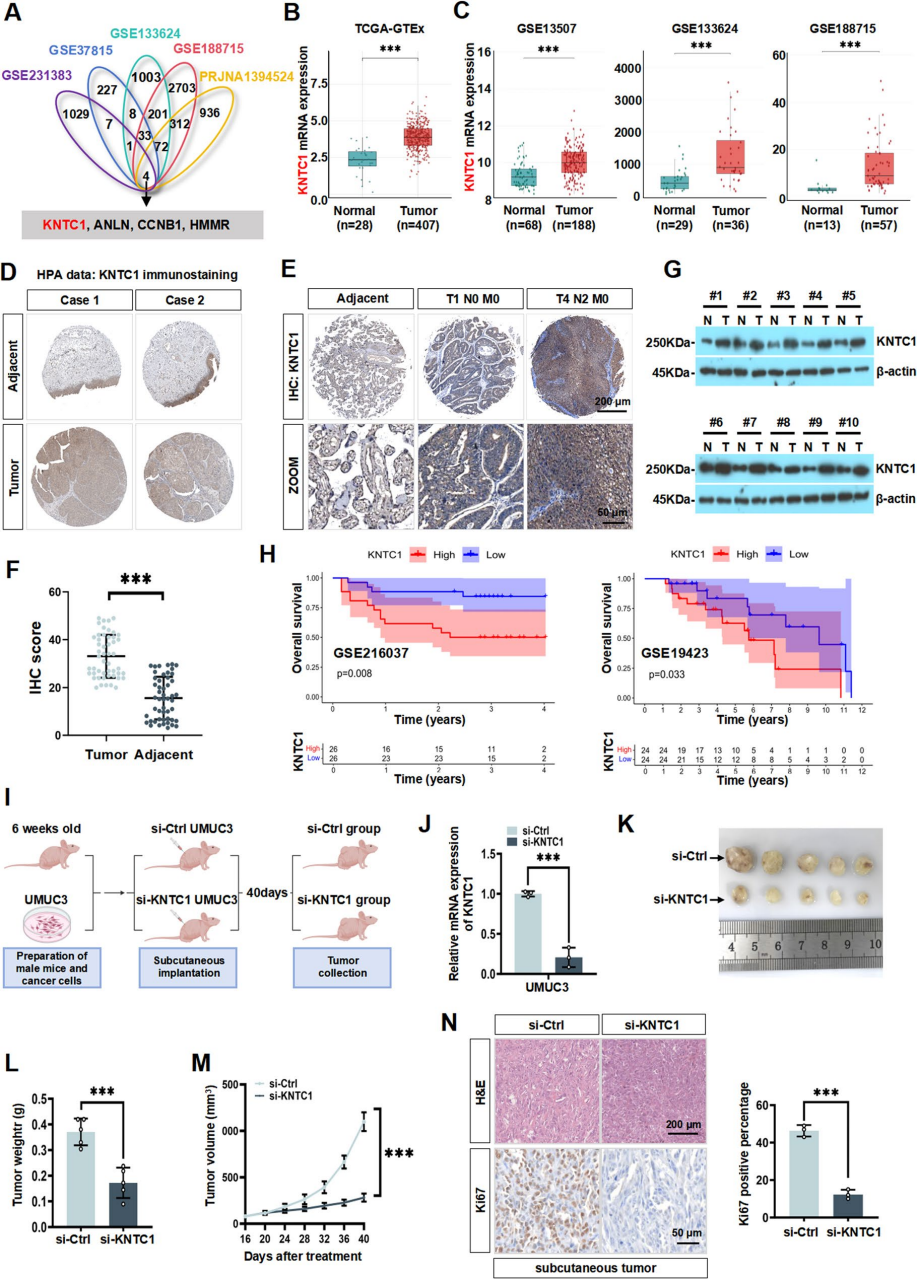
KNTC1 upregulation in BLCA associates with poor patient prognosis. **A** Venn diagram showing 4 potential oncogenes which cross-analyzed by three GEO databases (GSE133624, GSE188715, GSE231383, GSE37815) with our own sequencing data. **B **and** C** Analysis of *KNTC1* mRNA expression in normal bladder tissues and BLCA was performed using datasets from TCGA BLCA and GEO (GSE13507, GSE133624, GSE188715). **D** Representative immunohistochemistry images for normal bladder tissues and BLCA are shown from The Human Protein Atlas (HPA) database. **E** The expression of KNTC1 was detected by immunohistochemistry (IHC) in BLCA tissues and adjacent non-cancerous tissues. **F** A quantitative analysis of IHC scores was performed to determine the expression of KNTC1 in 50 pairs of clinical BLCA specimens we collected. **G** The protein expression level of KNTC1 was examined by western blotting in our collected BLCA tissues and paired adjacent normal tissues. **H** Kaplan-Meier overall survival (OS) curves for BLCA patients with high or low *KNTC1* mRNA expression from GEO datasets (GSE216037, GSE19432). **I** Schematic diagram of subcutaneous tumor model establishment and treatment. **J** The mRNA level of *KNTC1* was determined in sh-KNTC1 UMUC3 cells by RT-qPCR assay. **K** Representative images of subcutaneous xenograft tumors (*n* = 5 for each group). **L** Analysis of the tumour weight of the xenografts in each group. **M** Growth curves of the subcutaneous xenografts in each group. **N** Representative HE staining images (Scale bar = 200 µm) and IHC staining images (Scale bar = 50 µm) of Ki67 were presented in sh-Ctrl or sh-KNTC1 UMUC3 xenograft nude mice tissues. ns, no significance; * *P* < 0.05; ** *P* < 0.01; *** *P* < 0.001

To identify genes critically involved in BLCA progression, we integrated four public transcriptomic datasets (GSE231383, GSE37815, GSE133624, and GSE188715) with our paired RNA sequencing data from normal human ureteral epithelial cells (SV-HUC-1) and BLCA cells (UMUC3) (SRA: PRJNA1394524). This combined analysis revealed four consistently upregulated candidate genes in BLCA tissues: *KNTC1*, *ANLN*, *CCNB1* and *HMMR* (Fig. 1A). Further literature investigation revealed that the functions and mechanisms of ANLN [21], CCNB1 [22, 23], and HMMR [24] in BLCA have been relatively well documented. In contrast, although KNTC1 has been implicated in cancer promotion, its regulatory mechanisms, clinical significance, and role in chemoresistance specifically in BLCA remained unclear. Therefore, we selected KNTC1 for in-depth investigation. Consistent with its selection, analysis of the TCGA database and multiple GEO datasets confirmed that *KNTC1* mRNA expression was significantly upregulated in BLCA tissues (Fig. 1B and C), supporting its potential role as a potential driver gene in bladder cancer and warranting further mechanistic exploration. Meanwhile, we also found that the high level of *KNTC1* in BLCA was positively correlated with the tumorigenesis of stages 1, 2, 3, and 4 and metastasis status of BLCA, which based on the UALCAN database (Fig. S1A). Subsequently, to assess protein level of KNTC1 in BLCA, we initially examined publicly available immunohistochemical (IHC) staining results were obtained from the publicly accessible Human Protein Atlas (HPA) repository. Our analysis result confirmed significantly elevated KNTC1 protein expression in BLCA tissues compared to normal bladder tissues (Fig. 1D). We then selected 10 paired clinical specimens for western blotting analysis. The result confirmed significantly higher KNTC1 protein expression in tumor tissues compared to normal tissues (Fig. 1E and S1B). We further validated KNTC1 protein expression of 50 paired clinical BLCA specimens through IHC analysis. Representative IHC images from 3 paired cases clearly demonstrated stronger KNTC1 immunostaining in tumor tissues compared to adjacent bladder epithelium (Fig. 1F). The quantitative analysis of IHC scores across all 50 pairs confirmed significantly higher KNTC1 protein expression in tumors (Fig. 1G), consistent with the western blotting analysis. Given the observed upregulation of KNTC1 in BLCA, we next evaluated its potential prognostic significance. Survival analysis based on *KNTC1* mRNA expression levels was performed using data from the GEO datasets (GSE216037, GSE19423). Based on Kaplan-Meier survival analysis in GEO cohorts (GSE216037, GSE19423), individuals exhibiting elevated KNTC1 expression showed markedly reduced overall survival (OS) relative to patients with lower expression levels (Fig. 1H).

To investigate the function of KNTC1 in the tumorigenesis of BLCA, we utilized BALB/c nude mice bearing subcutaneous UMUC3 tumor-bearing models to investigate the in vivo antitumor efficacy of KNTC1. Briefly, UMUC3 tumor-bearing mice were randomly divided into two groups: one received peritumoral injection of in vivo RNA interfering system (siRNA, RiboBio) to knockdown of KNTC1 (si-KNTC1), and the other received non-targeting RNA interfering system as negative control (si-Ctrl) (Fig. 1I). The in vivo RNA interfering system was designed and synthesized by RiboBio Tech (Guangzhou, China). The in vivo RNA interfering system (si-KNTC1) effectively downregulated the expression of KNTC1 (Fig. 1J). Subsequently, we observed that si-KNTC1 group resulted in a more significant decrease in tumor size and weight compared to those from the si-Ctrl group (Fig. 1K and L). The inhibitory efficiency was calculated by the tumour weight (Fig. 1M). Furthermore, immunohistochemical (IHC) analysis demonstrated that si-KNTC1 treatment markedly reduced Ki67 expression levels in xenograft tumors (Fig. 1N). Collectively, these results suggested that KNTC1 is significantly overexpressed at both the mRNA and protein levels in BLCA and promotes tumor progression in vivo. Furthermore, the elevated expression of KNTC1 is strongly associated with poor clinical outcomes, specifically shorter overall survival. These findings highlight KNTC1 as a potential prognostic biomarker and therapeutic target in BLCA.

### KNTC1 promotes the proliferation, migration and invasion of BLCA cells in vitro

**Fig. 2:**
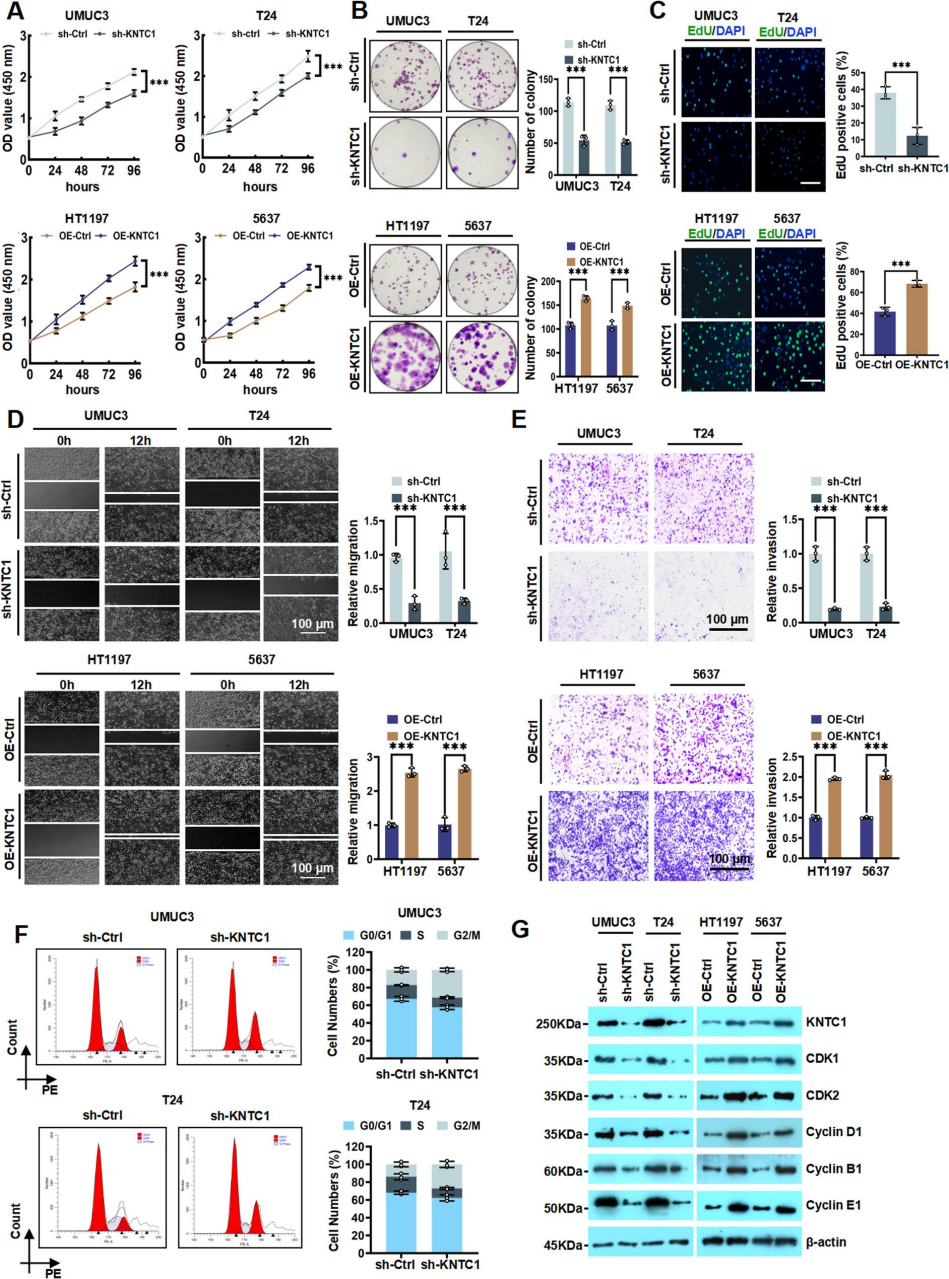
KNTC1 promotes the proliferation, migration and invasion of BLCA cells in vitro. **A** The effect of sh-KNTC1 and OE-KNTC1 on the proliferative ability of BLCA cells was evaluated by CCK-8 assay. **B** The colony formation assay was employed to validate the impact of sh-KNTC1 or OE-KNTC1 on the proliferationof various BLCA cells. **C** The EdU assay was utilized to detect the effects of sh-KNTC1 or OE-KNTC1 on BLCA cell proliferation. **D **and** E** The migratory and invasive capacities of BLCA cells with knockdown or overexpressed KNTC1 were assessed by wound healing assay (**D**) and transwell assay (**E**), respectively. **F** The cell cycle distribution was analyzed by flow cytometry after knockdown KNTC1 in UMUC3 and T24 cells. **G** The protein levels of the cell cycle-related proteins in sh-KNTC1 and OE-KNTC1 BLCA cells were evaluated using western blotting. ns, no significance; * *P* < 0.05; ** *P* < 0.01; *** *P* < 0.001

Comparative analysis across BLCA cell lines (5637, HT1197, SW780, J82, UMUC3, T24) and the human immortalized urothelial cell SV-HUC-1 confirmed elevated KNTC1 expression in all cancer cells (Fig. S2A and S2B). Specifically, the expression of KNTC1 was higher in T24 and UMUC3 cells and lower in 5637 and HT1197 cells. Therefore, we designed a lentiviral system to knock down KNTC1 (sh-KNTC1) in UMUC3 and T24 cells and to overexpress KNTC1 (OE-KNTC1) in 5637 and HT1197 cells to further explore the oncogenic potential of KNTC1 in vitro. The lentiviral vector system effectively downregulated or overexpressed the expression of KNTC1, as illustrated in Fig. S3A-S3D. Subsequent experiments using CCK-8 and colony formation assays demonstrated a decrease in cell growth rate in sh-KNTC1-treated UMUC3 and T24 cells. Conversely, OE-KNTC1 significantly enhanced cellular proliferation in HT1197 and 5637 cells (Fig. 2A and B). EdU assay further confirmed that sh-KNTC1 suppressed cellular proliferation by inhibiting DNA synthesis in UMUC3 and T24 cells, while OE-KNTC1 promoted proliferation in 5637 and HT1197 cells (Fig. 2C). Furthermore, transwell and wound healing assays indicated that sh-KNTC1 treatment significantly suppressed the invasive and migratory capacities of UMUC3 and T24 cells, whereas OE-KNTC1 treatment potentiated invasion and migration in HT1197 and 5637 cells (Fig. 2D-E), suggesting a potential function of KNTC1 in regulating cell invasion and migration. Moreover, due to KNTC1 is a centromere-related protein, we investigated the role of KNTC1 in cell cycle regulation. We found that knockdown of KNTC1 induced G2/M phase arrest (Fig. 2F). We further analyzed the correlation between the mRNA levels of KNTC1 and the mRNA levels of key cell cycle-related genes in BLCA cells, which revealed significant positive correlations (Fig. S4). Subsequently, western blotting analysis demonstrated that overexpression of KNTC1 upregulated the expression of cell cycle-associated proteins including CDK1, CDK2, cyclin D1, cyclin B1 and cyclin E1, whereas knockdown of KNTC1 downregulated their expressions (Fig. 2G; Fig. S5A and S5B). Collectively, these results established KNTC1 as an oncogenic driver regulating bladder cancer cell proliferation and invasion.

### KNTC1 promotes the malignant behaviour of BLCA cells via modulating PI3K/AKT/mTOR/MYC signaling pathway

**Fig. 3:**
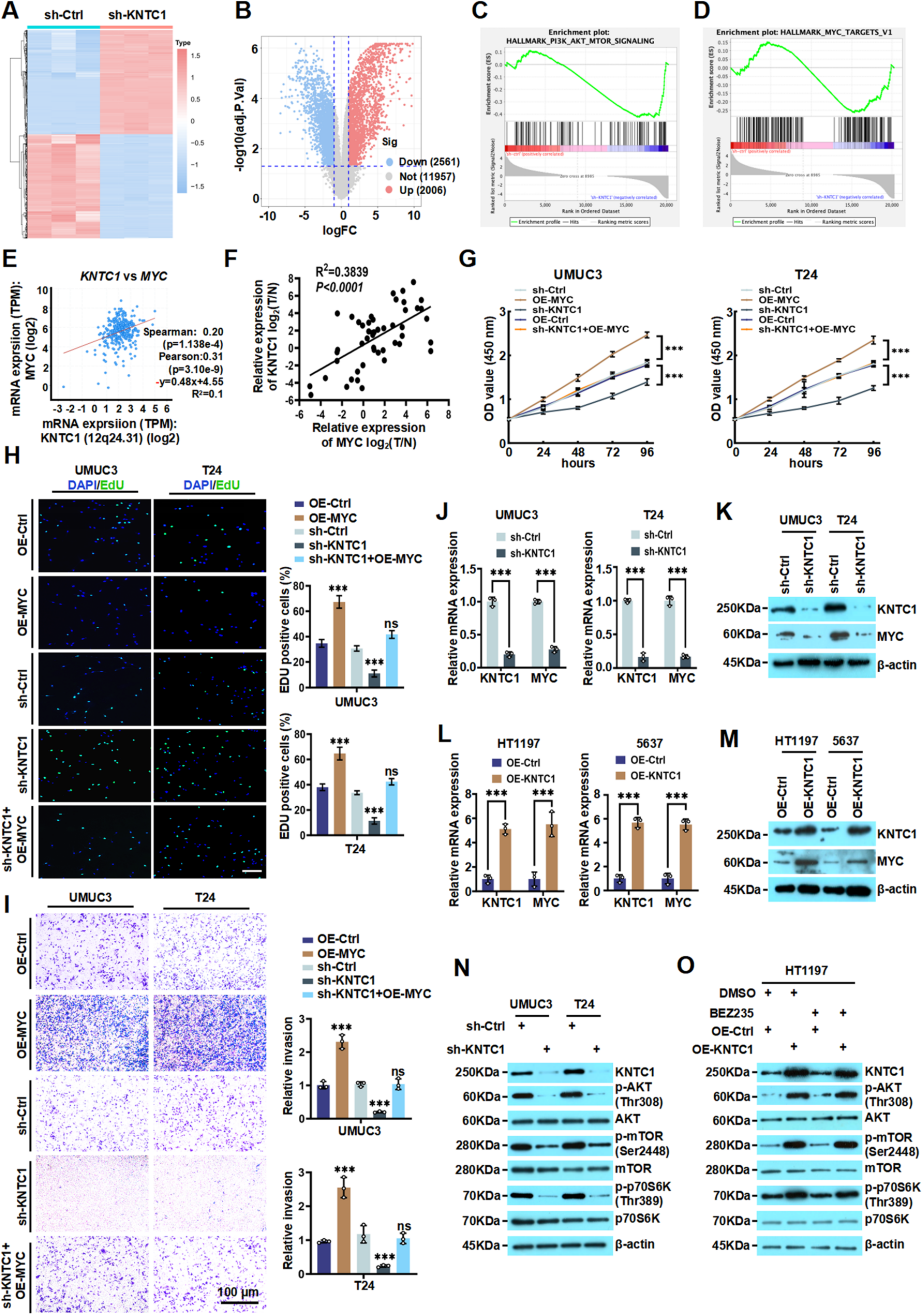
KNTC1 promotes the malignant behaviour of BLCA cells via modulating PI3K/AKT/mTOR/MYC signaling pathway.**A** Heatmap representing the expression levels of DEGs obtained from RNA sequencing of KNTC1 knockdown UMUC3 cells. Each column represents the indicated sample, and each row indicates one DEG. Red and blue colors indicate high or low expression, respectively. The expression value is shown as a Z score of the normalized transcripts per million (TPM). **B** The volcano plot shows the upregulated and downregulated DEGs in sh-KNTC1 UMUC3 cells compared to sh-Crtl UMUC3 cells. **C **and** D** Enrichment analysis of the high expression of the *KNTC1* gene of BLCA patients based on GSEA 4.2.3 software (https://www.gsea-msigdb.org/gsea/index.jsp). The HALLMARK_MYC_TARGETS_V1 gene set (NES = −1.67, NOM *p*-val < 0.001, FDR *q*-val = 0.003) and HALLMARK_PI3K_AKT_MTOR_SIGNALING (NES = −1.32, NOM *p*-val = 0.052, FDR *q*-val = 0.077). **E** Correlation analysis between *KNTC1* and *MYC* mRNA expression in the TCGA-BLCA cohort. **F** Analysis of the correlation between *MYC* and *KNTC1* mRNA expression in our collected BLCA patient samples. **G-I** CCK-8 assay (**G**), EdU assay (**H**), and Transwell invasion assay (**I**) demonstrate that OE-MYC can reverse the KNTC1 knockdown-induced inhibition of proliferation and invasion capacities of UMUC3/T24 cells. **J **and** K** The effects of KNTC1 knockdown on the expression of MYC was analyzed by RT-qPCR and western blotting, respectively. **L **and** M** The effects of OE-KNTC1 on the mRNA and protein levels of MYC were examined using RT-qPCR and western blotting, respectively. **N** Western blotting was performed to assess the effect of sh-KNTC1 on the phosphorylation levels of key proteins in the PI3K/AKT/mTOR pathway in UMUC3 and T24 cells. **O** The phosphorylation status of the PI3K/AKT/mTOR pathway after OE-MYC or treatment with the PI3K/AKT/mTOR inhibitor BEZ235 was examined by western blotting. ns, no significance; * *P* < 0.05; ** *P* < 0.01; *** *P* < 0.001

Although KNTC1 exhibits oncogenic properties in BLCA cell lines and xenografts, the downstream signaling mechanisms in BLCA cells remain unclear. To further elucidate the KNTC1-mediated regulatory network in BLCA cells, we performed RNA sequencing (RNA-seq) to analyze transcriptomic changes in UMUC3 cells treated with sh-KNTC1 or sh-Ctrl. By comparing the total mRNAs from sh-KNTC1-treated UMUC3 cells with those from sh-Ctrl cells, we identified 2006 upregulated DEGs (Up DEGs) and 2561 downregulated DEGs (Down DEGs; Fig. 3A). The heat map generated from the results revealed 2 hierarchical clusters based on similar gene expression profiles from 3 replicates under each condition (Fig. 3B). In GSEA analysis (Fig. 3C and D), the DEGs were predominantly enriched in pathways including “PI3K/AKT/mTOR signaling” (NES= −1.667, NOM *p*-val < 0.001, FDR *q*-val = 0.0030) and “MYC TARGETS_V1” (NES = −1.316, NOM *p*-val = 0.044, FDR *q*-val = 0.0470), underscoring the crucial role of KNTC1 in UMUC3 cells. Analysis of TCGA-BLCA data further demonstrated a positive association between *KNTC1* and *MYC* expression (Fig. 3E), which was corroborated in our own cohort of BLCA clinical specimens, where *KNTC1* mRNA levels correlated positively with those of *MYC* (Fig. 3F). To further study the relationship between KNTC1 and MYC in BLCA cells, we overexpressed MYC to conduct several rescue experiments in sh-KNTC1 BLCA cells. The suppressive effects of sh-KNTC1 on both proliferative and invasive capacities of UMUC3 and T24 cells were subsequently counteracted in the presence of OE-MYC (Fig. 3G and I). Meanwhile, we found knockdown of KNTC1 reduced both mRNA and protein levels of MYC, whereas OE-KNTC1 upregulated the expression of MYC (Fig. 3J and M; Fig. S6A and S6B). Previous studies have reported that MYC exerts its oncogenic functions in cancers by regulating the PI3K/AKT/mTOR pathway [25], we sought to investigate whether KNTC1 is involved in modulating MYC mediated by PI3K/AKT/mTOR pathway. In UMUC3 and T24 cells, we observed that knockdown of KNTC1 markedly reduced the phosphorylation levels of AKT (Thr308), mTOR (Ser2448), and p70S6K (Thr389) (Fig. 3N; Fig S7A and S7B). Furthermore, following treatment with the PI3K/AKT/mTOR pathway inhibitor BEZ235, overexpression of KNTC1 was able to rescue the suppressed phosphorylation of the PI3K/AKT/mTOR pathway (Fig. 3O and Fig S8A-S8C). Overall, these results indicated that KNTC1 may promote malignant behavior in BLCA cells by activating the PI3K/AKT/mTOR signaling pathway and regulating the expression of MYC on mRNA and protein levels.

### KNTC1 binds E2F8 and promotes E2F8 nuclear translocation to activate MYC transcription in BLCA

**Fig. 4:**
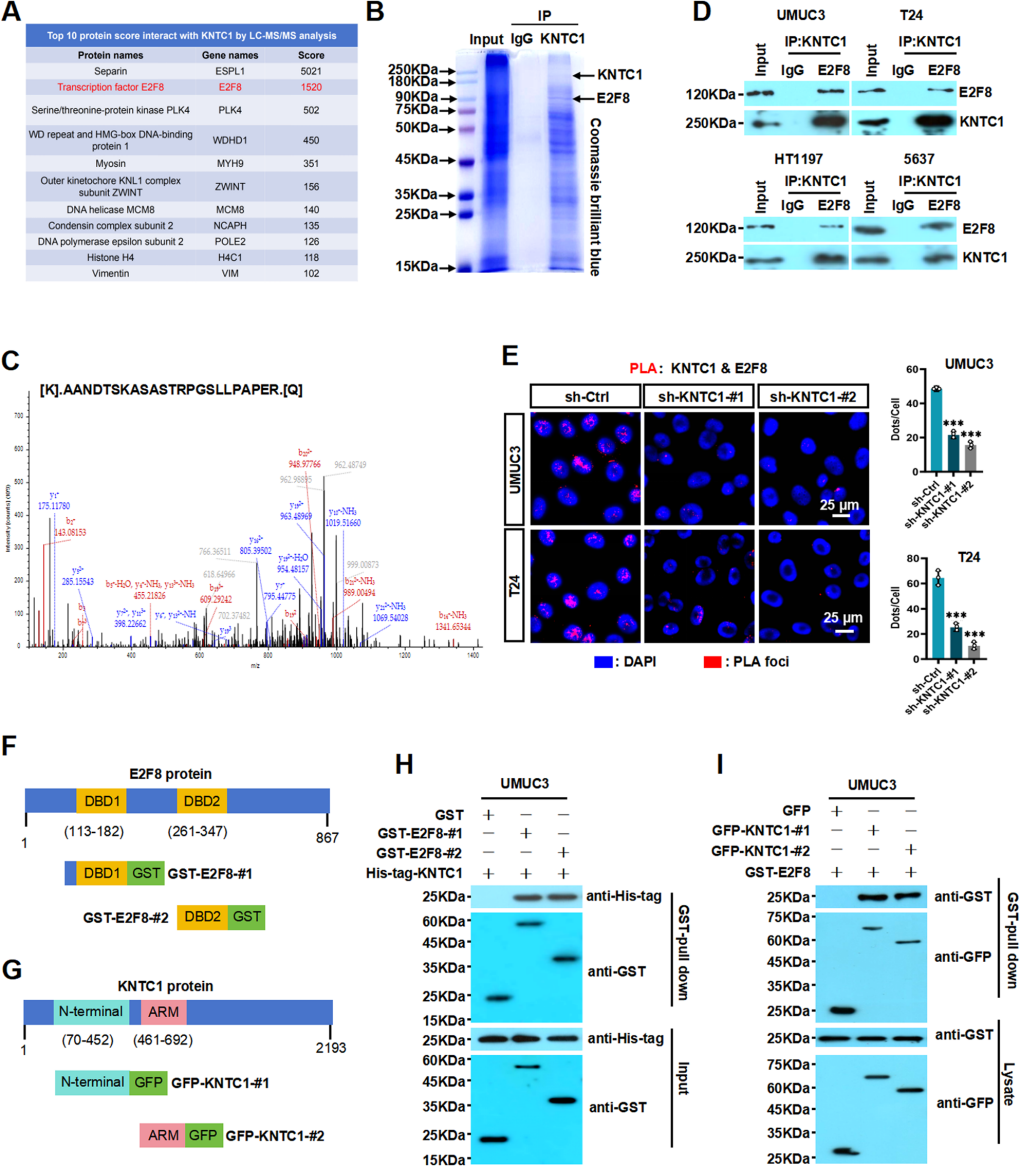
KNTC1 interacts with E2F8 in BLCA.**A** Screening for the top 10 protein score interact with KNTC1 in BLCA lines was performed using LC-MS/MS analysis. **B** The interaction between KNTC1 and E2F8 in UMUC3 cells was detected by Coomassie Brilliant Blue staining assay. **C** Mass spectrometry identification peak map of KNTC1-specific bands. **D** The specific interaction between KNTC1 and E2F8 in BLCA cells was confirmed by Co-IP assay. **E** Interaction of KNTC1 and E2F8 in UMUC3 and T24 cells visualized by the proximity ligation assay. **F **and** G** Schematic diagrams of the E2F8 truncations and the KNTC1 truncations. **H **and** I** GST pull-down assay. GST or GST fusion proteins were incubated with the indicated proteins and the bound proteins were analyzed by SDS-PAGE. GFP-tagged proteins and GST-tagged proteins were detected by Western blotting. ns, no significance; * *P* < 0.05; ** *P* < 0.01; *** *P* < 0.001

Our findings indicated that KNTC1 regulates the expression of MYC, no studies have reported that KNTC1 can directly regulate gene transcription. Therefore, we hypothesized that KNTC1 might cooperate with other certain transcription factors to achieve facilitate the activation of MYC transcription in BLCA cells. We then performed co-IP combined with liquid chromatography-tandem mass spectrometry (co-IP/LC-MS) assay to screen for KNTC1-interacting proteins. The results revealed that the transcription factor E2F8 is a prominent binding protein of KNTC1 (Fig. 4A). To further identify potential interacting proteins of KNTC1, we initially performed coomassie brilliant blue staining assay to validated the interaction between KNTC1 and E2F8 in UMUC3 cells. As shown in Fig. 4B, we found a distinct protein band specifically present at 90 kDa, which indicated that KNTC1 and E2F8 exist the physical interaction. Furthermore, E2F8 was identified by screening proteins associated with KNTC1 from the mass spectrometry results; the amino acid sequence of E2F8 as determined by mass spectrometry is shown in Fig. 4C. Meanwhile, the co-IP results further confirmed the specific interaction between KNTC1 and E2F8 in BLCA cells (Fig. 4D). Moreover, proximity ligation assay (PLA) also demonstrated that KNTC1 interacts with E2F8 in UMUC3 and T24 cells (Fig. 4E). We next sought to identify the regions of KNTC1 and E2F8 responsible for their specific interaction, we constructed a series of deletion mutants for both proteins (Fig. 4F and G). GST pulldown assays revealed that E2F8 fragments encompassing amino acids 113–182 and 261–347 failed to bind KNTC1, indicating that these two DNA-binding domains (DBDs) of E2F8 are necessary for the interaction in both HEK293 and UMUC3 cells (Fig. 4H and Fig. S9A). Conversely, the N-terminal region (amino acids 70–452) and the Armadillo (ARM) repeat domain (amino acids 461–692) of KNTC1 were found to interact with E2F8. These results suggest that both the N-terminal and ARM domains of KNTC1 are essential for its binding to E2F8 (Fig. 4I and Fig. S9B).

**Fig. 5:**
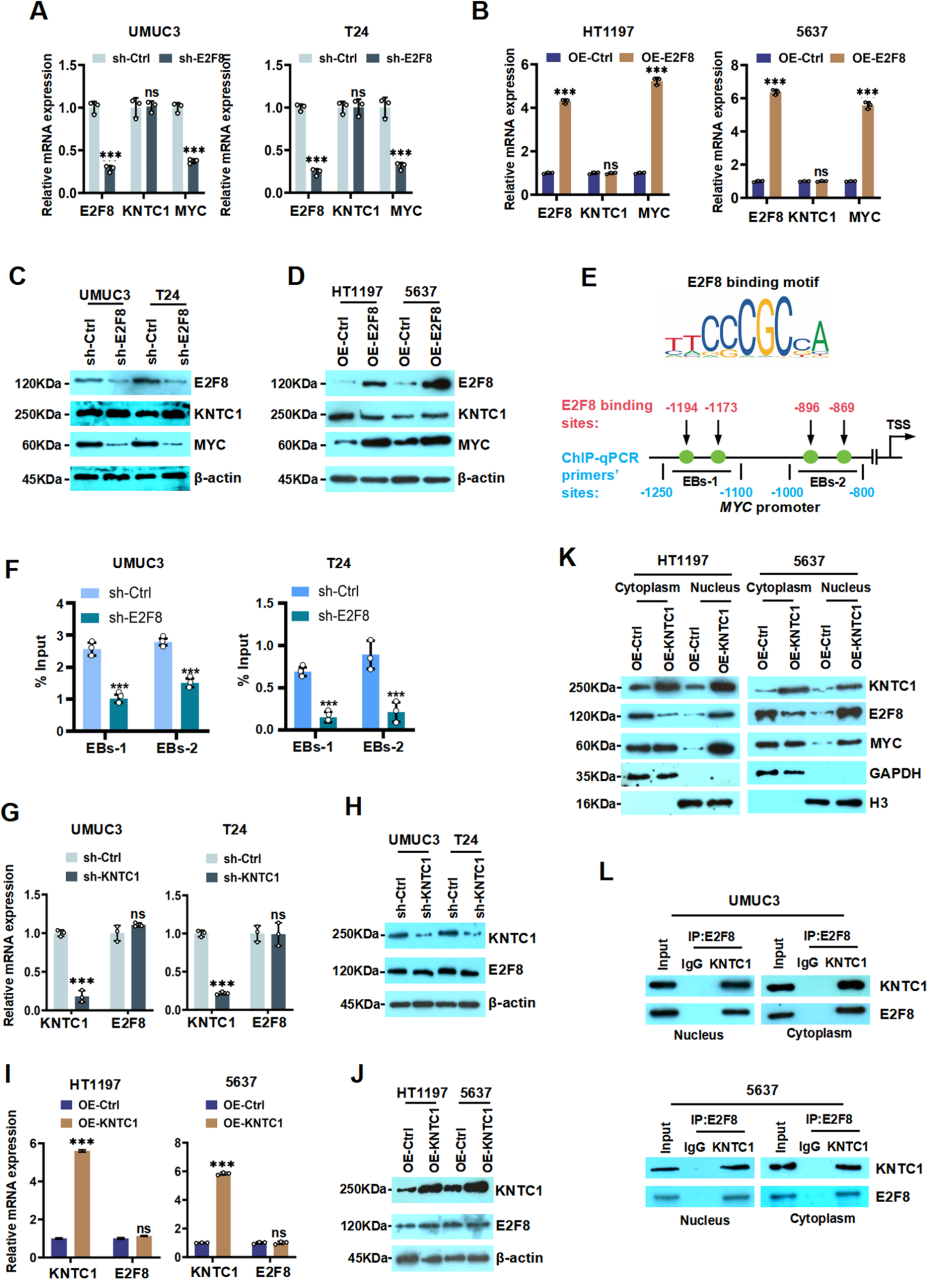
KNTC1 promotes E2F8 nuclear translocation to activate MYC transcription in BLCA.**A**-**D** RT-qPCR and western blotting results revealed the expression of KNTC1 and MYC in sh-E2F8/OE-E2F8 BLCA cells, respectively. **E** The E2F8 binding motif and E2F8 binding sites on MYC promoter were predicted using the JASPAR database. **F** The recruitment of E2F8 to the MYC promoter region in UMUC3 and T24 cells was analyzed by ChIP-qPCR. **G-J** The effects of KNTC1 knockdown or overexpressed on the mRNA and protein levels of E2F8 were analyzed by RT-qPCR and western blotting, respectively. **K** Western blotting demonstrated the expression of E2F8 in the nuclear and cytoplasm after OE-KNTC1. **L** The specific interaction between KNTC1 and E2F8 in BLCA cells’ cytoplasm and nucleus was confirmed by Co-IP assay. ns, no significance; * *P* < 0.05; ** *P* < 0.01; *** *P* < 0.001

We next investigated the effect of knockdown E2F8 on the expression of KNTC1 and MYC in BLCA cells. RT-qPCR and western blotting analyses revealed that E2F8 silencing significantly downregulated the expression of MYC at both mRNA and protein levels. Conversely, E2F8 overexpression robustly increased the expression of MYC, whereas the expression of KNTC1 remained unaffected under both conditions (Fig. 5A and D; Fig. S10A and S10B). We next sought to study the mechanism of E2F8 regulating *MYC* transcription in UMUC3 and T24 cells. We firstly predicted high-confidence binding motifs for E2F8 within the *MYC* promoter region (Fig. 5E) by JASPAR database. To validate these predicted binding sites, we performed ChIP assay as indicated. As shown in Fig. 5F, results of ChIP-qPCR demonstrated that E2F8 binds to the *MYC* promoter in UMUC3 or T24 cells. Collectively, our results indicated KNTC1 interacts with E2F8 to form a complex, while E2F8 could bind to the *MYC* promoter and activates *MYC* transcription in BLCA cells.

Since KNTC1 interacts with E2F8, we further investigated whether KNTC1 regulates E2F8 expression. RT-qPCR and western blotting analyses showed that neither knockdown nor overexpression of KNTC1 affected the total protein expression of E2F8 (Fig. 5G and I; Fig. S11A and S11B). However, nucleus-cytoplasm fractionation assay showed that OE-KNTC1 downregulated the expression of E2F8 in the cytoplasm whereas upregulated the expression of E2F8 in the nucleus, while the total protein level of E2F8 remained unchanged in BLCA cells (Fig. 5K; Fig. S12A and S12B). Furthermore, Co-IP assays were performed to examine the interaction between KNTC1 and E2F8 in subcellular locations. The results demonstrated that KNTC1 binds to E2F8 in both the cytoplasmic and nuclear fractions of BLCA cells (Fig. 5L). These results indicated that KNTC1 regulating the nucleus translocation of E2F8, thereby potentiating E2F8-mediated transcriptional activation of MYC. Collectively, our findings demonstrated that KNTC1 directly interacts with the transcription factor E2F8 and promotes its nuclear accumulation without altering E2F8 total expression. Subsequently, this redistribution enhances E2F8 binding to the *MYC* promoter and facilitates *MYC* transcriptional activation. Thus, KNTC1 acts as a critical upstream regulator that orchestrates the E2F8/MYC axis through controlling E2F8 subcellular localization, revealing a novel mechanism that contributes to BLCA malignancy.

### E2F8 drives BLCA progression in vitro and in vivo

**Fig. 6:**
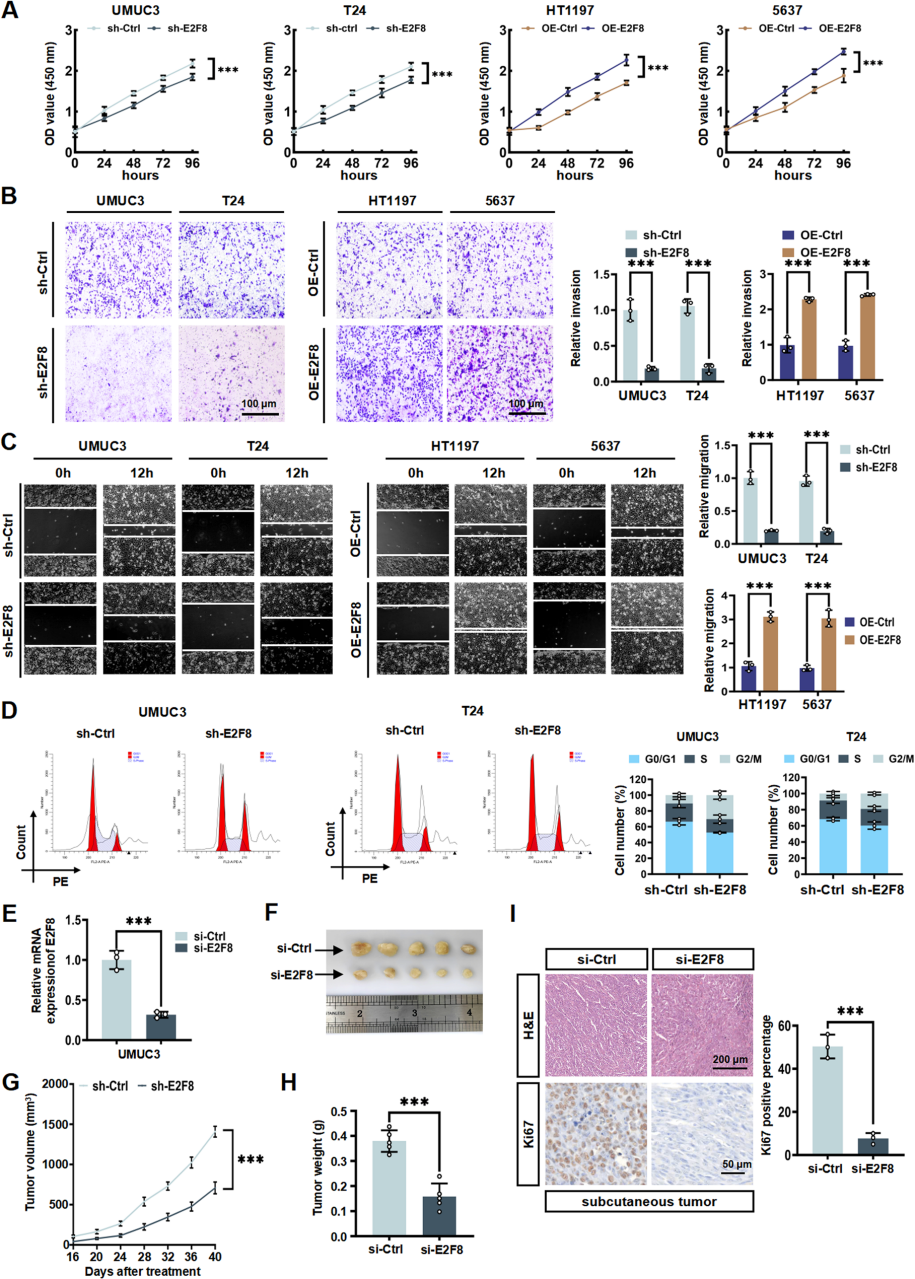
E2F8 drives BLCA progression in vitro and in vivo. **A** After knocking down E2F8 in UMUC3/T24 cells and overexpressing E2F8 in HT1197/5637 cells, the cell proliferation was evaluated by the CCK-8 assay. **B** Transwell experiment was conducted to measure the invasion ability of UMUC3/T24 cells after knocking down E2F8 and HT1197/5637 cells after overexpression of E2F8. **C** The migration ability of UMUC3 and T24 cells treated with sh-E2F8 and HT1197 and 5637 cells treated with OE-E2F8 were evaluated through the wound healing experiment. **D** The cell cycle of UMUC3 and T24 cells treated with sh-Ctrl and sh-E2F8 were analyzed by flow cytometry. **E** E2F8 knockdown efficiency in UMUC3 and T24 cells was measured by RT-qPCR. **F** Representative images of subcutaneous xenograft tumors obtained from nude mice after knockdown of E2F8. **G** Growth curves of subcutaneous xenograft tumors in each group. **H** Tumor weight analysis of xenograft tumors in each group. **I** Representative HE staining (scale = 200 micrometers) images and IHC staining images (scale = 50 micrometers), respectively showing the tissues of subcutaneous xenograft nude mice in the control group or those after knockdown of E2F8. ns indicates no significant difference; * *P* < 0.05; ** *P* < 0.01; *** *P* < 0.001

As a member of the E2F transcription factor family located on chromosome 11p15.1, E2F8 has been reported to promote tumorigenesis in various cancers by regulating cell cycle progression and proliferation [26–28]. More recently, Liu et al. reported that E2F8 is a potential oncogenic factor for the progression of BLCA [29]. Next, we aimed to further validate the role of E2F8 in BLCA cells. To assess E2F8’s impact on BLCA cell proliferation, we performed CCK-8 assays on knocking down of E2F8 (sh-E2F8) and overexpression of E2F8 (OE-E2F8) cells (Fig. 6A). The results showed that sh-E2F8 inhibited BLCA cell proliferation, while overexpression reversed it. Transwell assays demonstrated that E2F8 depletion suppressed invasion and migration capabilities in UMUC3 and T24 cells (Fig. 6B). Meanwhile, wound healing assays revealed impaired migration upon sh-E2F8 (Fig. 6C). Conversely, OE-E2F8 promoted invasion and migration in HT1197 and 5637 cells. These findings demonstrated that E2F8 enhances the proliferation, invasion, and migration capacities of BLCA cells. Furthermore, knockdown of E2F8 inhibited G2/M phase progression (Fig. 6D). To investigate the tumorigenic function of E2F8 in vivo, subcutaneous xenograft tumors were generated in BALB/c nude mice using UMUC3 cells. Mice bearing established tumors were randomized into two treatment groups: one receiving peritumoral injections of si-Ctrl and the other receiving si-E2F8. RT-qPCR confirmed that in vivo system of si-E2F8 effectively knocked down E2F8 expression in the xenograft tumor (Fig. 6E). Post-intervention analysis revealed significantly reduced tumor weight and size in the si-E2F8 group relative to the si-Ctrl group (Fig. 6F and G). Based on tumor weight, E2F8 knockdown significantly inhibited tumor growth (Fig. 6H). Additionally, IHC staining showed that si-E2F8 treatment substantially lowered Ki67 expression levels in the xenograft tumors (Fig. 6I). Collectively, our findings indicate that E2F8 facilitates the growth of BLCA cells in vitro and in vivo.

### MYC promotes KNTC1 transcription in BLCA cells

**Fig. 7:**
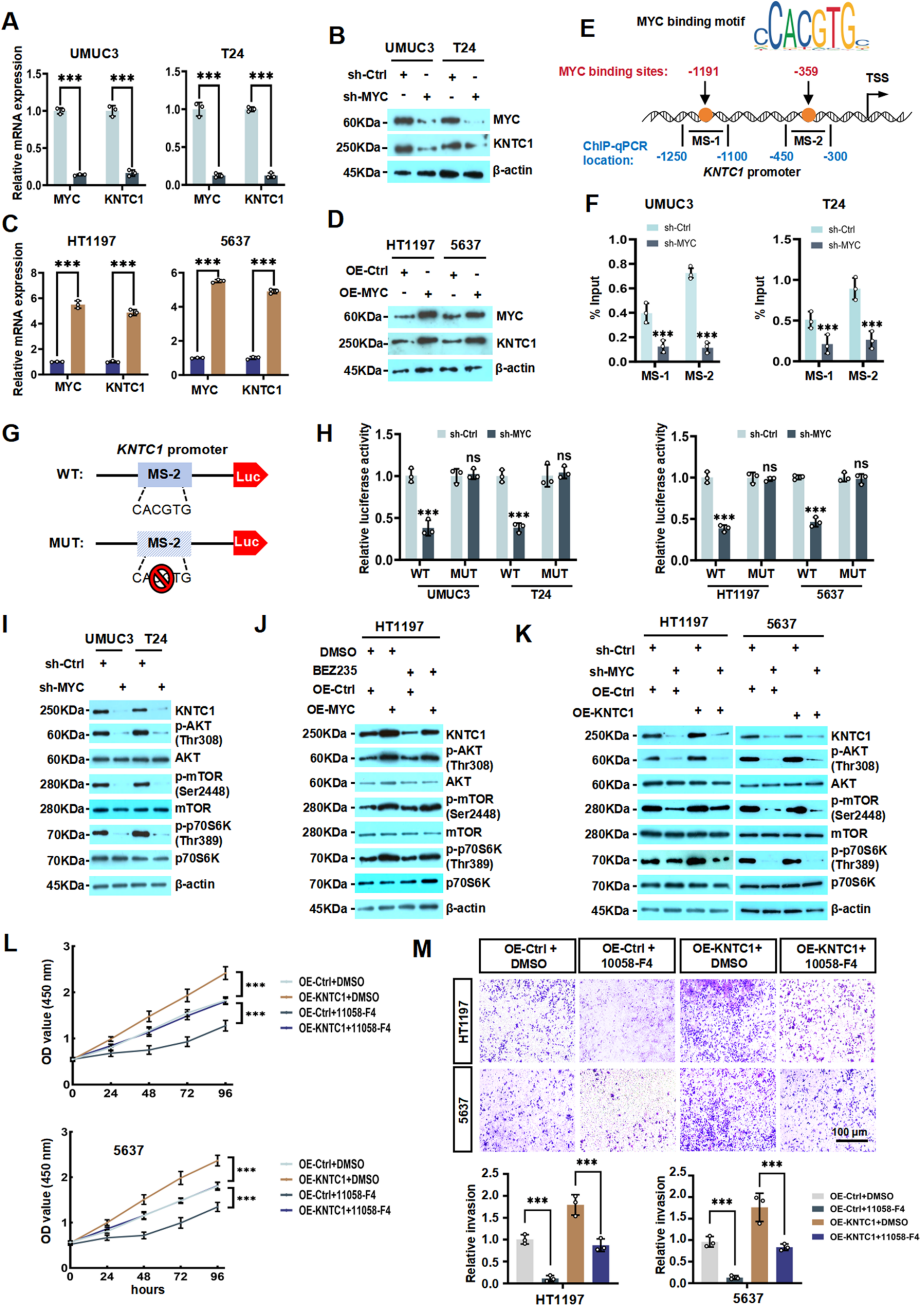
MYC promotes KNTC1 transcription in BLCA cells.**A** RT-qPCR analysis of *KNTC1* mRNA expression following MYC knockdown in UMUC3 and T24 cells. **B** Western blotting analysis revealed the protein expression of KNTC1 was observed in UMUC3 and T24 cells after knockdown MYC. **C** RT-qPCR assay showed that OE-MYC significantly enhanced the mRNA level of *KNTC1* in UMUC3 and T24 cells. **D** Western blot analysis demonstrated the protein level of KNTC1 in UMUC3 and T24 cells after knockdown of the *MYC* gene. **E** Schematic diagram of the location of the *MYC* binding site to the promoter of the *KNTC1* gene. **F** ChIP-qPCR analyzed the enrichment of *MYC* on the *KNTC1* promoter in UMUC3 and T24 cells treated with sh-Ctrl or sh-MYC. **G **and** H** Dual luciferase reporter gene assay compared the activity of the reporter gene in the control group and WT-*KNTC1* in sh-MYC cells with that in MUT-*KNTC1* under the same conditions. **I** Western blotting showed that after knockdown of MYC in UMUC3 and T24 cells, the protein expressions of KNTC1, p-AKT, AKT, p-mTOR, mTOR, p-p70S6K, and p70S6K were observed. **J** Western blotting also showed that after overexpression of MYC or treatment with the PI3K/AKT/mTOR inhibitor BEZ235, the protein expressions of KNTC1, p-AKT, AKT, p-mTOR, mTOR, p-p70S6K, and p70S6K were detected. **K** Western blotting also showed that the protein expressions of KNTC1, p-AKT, AKT, p-mTOR, mTOR, p-p70S6K, and p70S6K were observed after knockdown of MYC or overexpression of KNTC1. **L **and** M** The effect of OE-KNTC1 and 10,058-F4 on the proliferative and invasive ability of BLCA cells was evaluated by CCK-8 and transwell assay. ns, no significant difference; * *P* < 0.05; ** *P* < 0.01; *** *P* < 0.001

Based on the established role of KNTC1 as a critical regulator of malignancy in BLCA, we next sought to elucidate the upstream molecular mechanisms governing its expression. MYC is a well-characterized transcription factor known to activate numerous downstream targets that drive oncogenesis, including in BLCA [30]. Notably, we demonstrated that KNTC1 collaborates with MYC in BLCA cells. On the basis of this functional interplay, we investigated that whether MYC regulates the transcription of *KNTC1* in BLCA cells. Intriguingly, we knocked down MYC (sh-MYC) in UMUC3 and T24 cells and found that KNTC1 expression was significantly downregulated at both the transcriptional and protein levels (Fig. 7A and B; Fig. S13A). Similarly, when we overexpressed MYC (OE-MYC) in 5637 and HT1197 cells, KNTC1 expression was significantly upregulated (Fig. 7C and D; Fig. S13B). To further investigate the mechanism by which MYC regulates *KNTC1* transcription, we then screened the JASPAR database and identified several potential binding motifs for MYC on the *KNTC1* promoter and validated it by ChIP-qPCR in UMUC3 and T24 cells. Subsequently, we found that the MYC was significantly enriched in the predicted *KNYC1* promoter regions (MS-1 or MS-2) compared to IgG controls. These findings indicated that *KNTC1* is transcriptionally regulated by MYC in UMUC3 and T24 cells (Fig. 7E and F). To further validate the regulatory mechanism between MYC and KNTC1, we performed a dual-luciferase reporter assay. Plasmids harboring the wild-type (WT-KNTC1) and mutated (MUT-KNTC1) forms of KNTC1 were constructed based on the predicted binding sequence (MS-2) between KNTC1 and MYC (Fig. 7G). Quantitative luciferase assays (Fig. 7H) revealed that WT-KNTC1 exerted significant transcriptional repression in sh-MYC cells compared to controls, whereas MUT-KNTC1 showed no significant regulatory effect on reporter activity under the same conditions. These results further confirmed that MYC transcriptionally activates KNTC1 in BLCA cells.

Next, we also study whether MYC regulating KNTC1 mediated by PI3K/AKT/mTOR signaling pathway. We found that knockdown of MYC in UMUC3 and T24 cells suppressed phosphorylation levels of the AKT (Thr308), mTOR (Ser2448), and p70S6K (Thr389) (Fig. 7I; Fig. S14). Moreover, OE-MYC reversed the inhibitory effect of the PI3K/AKT/mTOR inhibitor BEZ235 on the PI3K/AKT/mTOR pathway (Fig. 7J; Fig. S15). More importantly, we also found that overexpression of KNTC1 rescued the suppression of PI3K/AKT/mTOR phosphorylation induced by sh-MYC (Fig. 7K; Fig. S16). Furthermore, we observed that 10,058-F4 (MYC inhibitor) rescued the oncogene effects induced by OE-KNTC1 in BLCA cells, further indicating that MYC can regulate KNTC1 expression (Fig. 7L and M). Overall, our current findings demonstrated that KNTC1 as a new downstream target of MYC in BLCA cells, collectively enhancing the phosphorylation of the PI3K/AKT/mTOR pathway to exert oncogenic functions.

### Silencing KNTC1 and MYC inhibits growth of gemcitabine resistant BLCA cells in vitro and in vivo

**Fig. 8:**
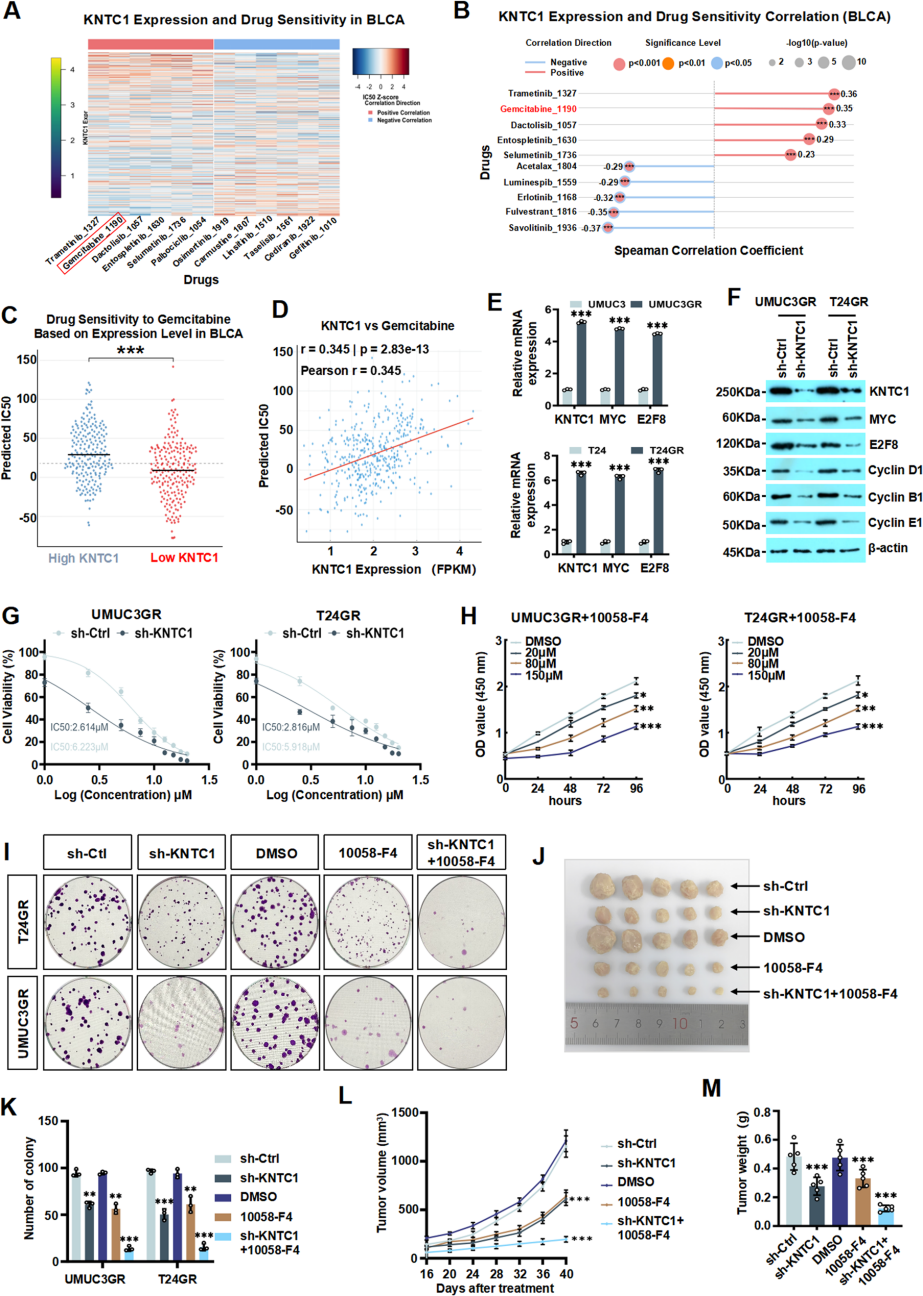
Silencing KNTC1 and MYC inhibits growth of gemcitabine resistant BLCA cells in vitro and in vivo. **A** The heatmap shows the correlation between *KNTC1* RNA level and sensitivity to different drugs based on TCGA and GDSC data. **B** The stick plot shows the correlation between the RNA level of *KNTC1* and various drug sensitivities, where the size of the stick figure represents the magnitude of the correlation. **C** The scatter plot shows the difference (*P* value) in the IC50 of gemcitabine between high *KNTC1* expression samples and low *KNTC1* expression samples in BLCA data. **D** The correlation scatter plot shows the correlation between the RNA expression of *KNTC1* and the IC50 of gemcitabine (Pearson coefficient). **E** RT-qPCR verified the mRNA levels of *KNTC1*, *MYC*, and *E2F8* in UMUC3, UMUC3GR, T24 and T24GR cells. **F** Western blotting showed the protein expression of KNTC1, MYC, E2F8, Cyclin D1, Cyclin B1, and Cyclin E1 in UMUC3GR/T24GR cells after knockdown of KNTC1. **G** IC50 assays were performed on sh-KNTC1 gemcitabine-resistant BLCA cells to evaluate the effect of sh-KNTC1 on the gemcitabine sensitivity of UMUC3GR or T24GR cells. **H** CCK8 experiments showed the changes in cell proliferation ability after adding different concentrations of 10,058-F4 to UMUC3GR cells and T24GR cells. **I **and** J** Colony formation experiments verified the colony formation ability in different treatment groups: knockdown of KNTC1, addition of 10,058-F4, knockdown of KNTC1 and combined treatment. **K** Representative images of subcutaneous xenograft tumors obtained from nude mice after treatment with knockdown of KNTC1, addition of 10,058-F4, and combined treatment with knockdown of KNTC1 and 10,058-F4 on UMUC3 cells. **L** Growth curves of subcutaneous xenograft tumors in each group. **M** Tumor weight analysis of subcutaneous xenograft tumors in each group.ns indicates no significant difference; * *P* < 0.05; ** *P* < 0.01; *** *P* < 0.001

Gemcitabine (Gem), a pyrimidine nucleoside analog, is widely used as an intravesical chemotherapeutic agent for both non-muscle-invasive and muscle-invasive bladder cancer (NMIBC and MIBC), demonstrating considerable efficacy and a favorable safety profile [31]. Compared with mitomycin, gemcitabine has been associated with superior outcomes in terms of reducing postoperative recurrence and extending progression-free survival in bladder cancer patients [32]. Additionally, inhibition of the PI3K/AKT/mTOR/MYC signaling pathway has been shown to enhance gemcitabine sensitivity in BLCA [33–38]. Therefore, we next aimed to investigate whether KNTC1 is involved in regulating the sensitivity of BLCA to chemotherapeutic Gem. To explore the link between KNTC1 expression and chemoresistance in BLCA, and its potential mechanisms, we acquired transcriptomic data for TCGA-BLCA from the UCSC XENA portal. Utilizing the GDSC drug database, we applied the “calcPhenotype” algorithm from the “oncoPredict” package to evaluate drug response phenotypes. Our analysis indicated a significant correlations between KNTC1 expression and sensitivity to Gem (Fig. 8A and D). We assessed the expression of KNTC1, MYC, E2F8 in Gem-resistant UMUC3 and T24 cells (UMUC3/GR and T24/GR), revealing significant upregulation compared to normal cells (Fig. 8E; Fig. S17A and S17B). Furthermore, we examined the effect of *KNTC1* knockdown on cell cycle-related markers in these Gem-resistant cells. As shown in Fig. 8F and Fig. S18, we found that silencing KNTC1 markedly decreased the expression of these markers. To evaluate the effect of KNTC1 on Gem sensitivity in UMUC3/GR or T24/GR cells, we performed IC50 assays on sh-KNTC1 Gem-resistant BLCA cells. We found that BLCA cells with konckdown KNTC1 expression exhibited significantly higher IC50 values for Gem, indicating KNTC1 enhanced Gem resistance in UMUC3/GR and T24/GR cells (Fig. 8G). Moreover, inhibiting MYC expression is a potential therapeutic strategy to overcome Gem resistance in BLCA cells [37, 38]. We next investigated the effect of combining KNTC1 knockdown with treatment of the 10,058-F4 [39, 40] on the proliferation of Gem-resistant cells. We treated UMUC3/GR or T24/GR cells with five groups: DMSO control, sh-Ctrl, sh-KNTC1, 10,058-F4 and a combination of sh-KNTC1 with 10,058-F4, respectively. Colony formation assays demonstrated that treatment with 10,058-F4 significantly reduced Gem-resistant cell growth compared to the control group. Notably, the combination of sh-KNTC1 and 10,058-F4 treatment significantly suppressed colony formation compared to 10,058-F4 treatment alone (Fig. 8H and I). To assess the impact of combination sh-KNTC1 and 10,058-F4 on Gem-resistant BLCA cells in vivo, nude mice were subcutaneously injected with sh-Ctrl and sh-KNTC1 T24/GR cells and divided into five groups: DMSO, sh-Ctrl, sh-KNTC1, 10,058-F4 and 10,058-F4 + sh-KNTC1, respectively (Fig. 8J). The combination of sh-KNTC1 and 10,058-F4 treatment significantly reduced tumor growth rate and tumor weight compared to other treatment groups (Fig. 8K and M). Taken together, to our knowledge for the first time, our data revealed that KNTC1 upregulation promotes Gem resistance in BLCA. Knocking down KNTC1, especially when combined with a MYC inhibitor, overcomes this resistance and suppresses tumor cell growth both in vitro and in vivo.

### A Poly(ß-amino ester) nanocarrier delivering sh-KNTC1 to inhibit the growth of distant metastatic Gem-resistant BLCA cells

Given the crucial role of KNTC1 upregulation in BLCA, we aimed to construct an efficient shRNA delivery vehicle targeting KNTC1 for potential therapeutic application. Previous findings confirmed that the sh-KNTC1 system (pRNAT-U6.1/Neo-KNTC1, Fenghbio, Changsha, China) efficiently downregulated KNTC1 expression and impeded cellular proliferation, migration, and invasion in vitro. We next extended our investigation to evaluate the influence of KNTC1 silencing on tumor proliferation and metastatic spread in vivo.

**Fig. 9:**
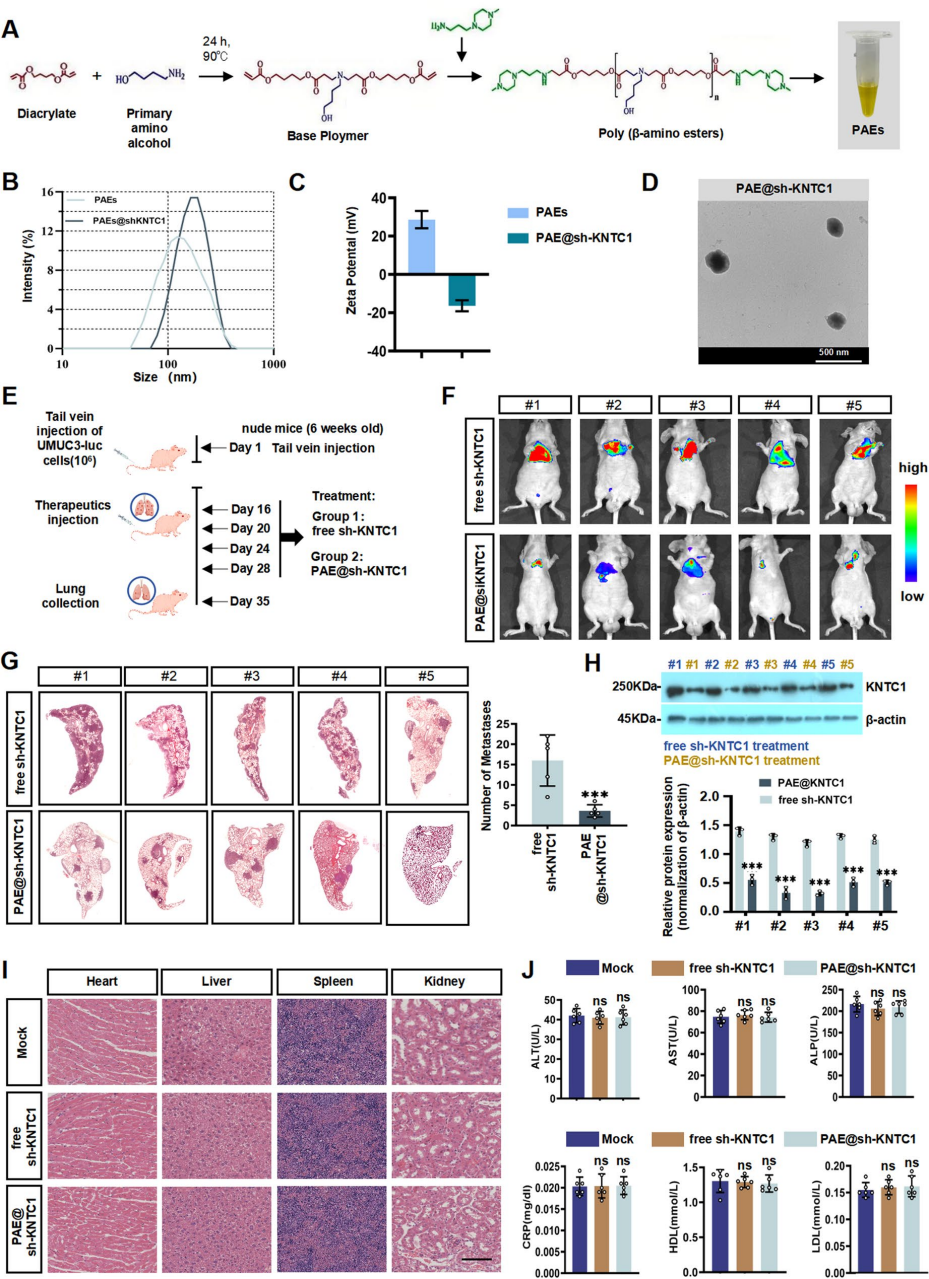
A Poly(ß-amino ester) nanocarrier delivering sh-KNTC1 to inhibit the growth of distant metastatic Gem-resistant BLCA cells.**A** Schematic diagram of the stepwise preparation of PAEs. **B** The average diameter (nm) of un-loaded PAE and PAE bound to sh-KNTC1. **C** Zeta potential of PAEs and PAEs loaded with sh-KNTC1. **D** Images of PAEs not loaded and PAEs bound to sh-KNTC1 under an electron microscope. **E** Schematic diagram of lung metastasis model establishment and treatment. **F** The representative images of IVIS imaging reveal the inhibitory effect of free sh-KNTC1 and PAE-loaded sh-KNTC1 treatment on lung metastasis of UMUC3-luc cells in vivo. **G** The representative image of HE staining of mouse lung sections reveals the metastatic nodules that appeared after treatment and analyzes the number of metastatic nodules in the lungs of nude mice. **H** Western blotting showed the protein expression of KNTC1 in UMUC3-luc cells under PAE@sh-KNTC1 treatment. **I** Representative images of HE staining of lung metastatic nodules in each group. **J** Blood biochemical analysis. ns indicates no significant difference; * *P* < 0.05; ** *P* < 0.01; *** *P* < 0.001

A class of poly(ß-amino ester)s (PAEs) was synthesized to serve as a carrier for sh-KNTC1 delivery [41]. The branched PAEs were prepared through a Michael addition reaction involving amines and acrylates, with glycerol triacrylate acting as a cross-linker [41]. The resulting PAE solution exhibited a light-yellow color (Fig. 9A), with average diameters of approximately 200 nm for unloaded PAEs and 300 nm for PAEs complexed with sh-KNTC1 (Fig. 9B). Successful encapsulation of sh-KNTC1 via electrostatic binding was verified by zeta potential assessment [42, 43]. As indicated in Fig. 8C, the zeta potential decreased from + 33.5 mV for free PAEs to −18.5 mV after complexation with sh-KNTC1 (PAE@sh-KNTC1) (Fig. 9C), confirming the formation of PAE@sh-KNTC1 nanoparticles. Morphological features were further examined using transmission electron microscopy (Fig. 9D).

To evaluate the anti-tumor efficacy of PAE@sh-KNTC1 in vivo, a metastatic BLCA model was established in nude mice (Fig. 9E). After 2 weeks of tail vein injection of UMUC3-luc cells, the animals received tail vein injections of either free shRNA or PAE@sh-KNTC1 (40 µg shRNA plasmid equivalent per mouse). Tumor development was monitored via bioluminescence imaging (BLI). Representative images in Fig. 9F illustrate lung metastases, with noticeably reduced fluorescence signal intensity in the PAE@sh-KNTC1 group relative to free shRNA. Correspondingly, HE staining of lung tissues revealed a lower number of metastatic nodules following PAE@sh-KNTC1 administration compared with controls (Fig. 9G).

To confirm that the antitumor response was specifically due to KNTC1 knockdown, expression levels were assessed in tissues treated with free sh-KNTC1 versus PAE@sh-KNTC1. As shown in Fig. 9H, marked reduction of KNTC1 was observed exclusively in the PAE@sh-KNTC1 group, supporting sequence-specific gene silencing. Furthermore, HE staining of major organs (Fig. 9I) and serum biochemical parameters (Fig. 9J) revealed no significant signs of toxicity, indicating a favorable safety profile. Collectively, these results demonstrate that PAE@sh-KNTC1 effectively inhibits pulmonary metastasis of BLCA in nude mice.

## Discussion

Despite advances in treatment, BLCA remains a malignancy with high rates of recurrence and chemoresistance, underscoring the need for novel therapeutic targets and a deeper understanding of its molecular drivers. In this study, we identify KNTC1 as a critical oncoprotein that is significantly upregulated in BLCA and correlates with poor patient prognosis. More importantly, we elucidate a previously unrecognized positive feedback loop between KNTC1 and the well-known oncogene MYC, mediated through the transcription factor E2F8 and the PI3K/AKT/mTOR pathway. This self-reinforcing signaling axis not only promotes tumor proliferation and metastasis but also confers gemcitabine resistance, positioning KNTC1 as a central node in BLCA pathogenesis and a promising therapeutic target.

Our findings significantly extend the current understanding of KNTC1’s function beyond its canonical role in mitosis and chromosome segregation. While previous studies have primarily focused on KNTC1’s function in the mitotic checkpoint [20], our work is the first to comprehensively demonstrate its potent non-mitotic, pro-tumorigenic functions in BLCA. We provide robust evidence that KNTC1 drives proliferation, invasion, and cell cycle progression in vitro and tumor growth in vivo. This establishes KNTC1 not merely as a bystander in cell division but as a bona fide oncogenic driver in BLCA, a conceptual advance that differentiates our work from prior research [23].

The most innovative aspect of our study is the discovery of the KNTC1-E2F8-MYC regulatory circuit. We found that KNTC1 does not directly regulate transcription but instead performs a novel “chaperone-like” function by binding to E2F8 and facilitating its nuclear translocation. This mechanism enhances E2F8-mediated transcriptional activation of MYC, thereby directly linking a core mitotic apparatus component to a master regulator of oncogenesis. Crucially, we also demonstrate that MYC, in turn, transcriptionally upregulates KNTC1, creating a powerful positive feedback loop that amplifies oncogenic signaling. This reciprocal regulation provides a novel explanation for the sustained overexpression of both molecules in BLCA, which has previously been attributed to separate mechanisms. Our ChIP and luciferase reporter assays conclusively validate this direct regulatory relationship. Furthermore, we place this novel axis within the context of the well-established PI3K/AKT/mTOR pathway. Our data suggest that the KNTC1-E2F8-MYC loop both influences and is reinforced by this pathway. KNTC1 knockdown suppressed PI3K/AKT/mTOR activation, while MYC overexpression could rescue the pathway inhibition caused by KNTC1 knockdown, and vice versa. This intricate crosstalk suggests that KNTC1 is a key upstream modulator that orchestrates a signaling network central to BLCA malignancy, offering a more integrated view of its pathophysiology.

From a translational perspective, our work unveils KNTC1’s critical role in gemcitabine resistance. The correlation between high KNTC1 expression and gemcitabine sensitivity in bioinformatics analyses, combined with our functional validation showing that KNTC1 knockdown resensitizes resistant cells, directly addresses a major clinical challenge in BLCA management. The most compelling therapeutic evidence comes from our in vivo combination therapy, where the synergistic effect of KNTC1 knockdown and MYC inhibition outperformed either monotherapy. This suggests that simultaneously targeting multiple nodes within this self-reinforcing loop could be a highly effective strategy to overcome chemoresistance. Finally, we successfully translated these findings into a potential therapeutic modality by developing a biocompatible poly (ß-amino ester) nanocarrier [44–46]. This system (PAE@sh-KNTC1) efficiently delivered sh-KNTC1 in vivo, significantly inhibiting lung metastasis without detectable toxicity, thereby providing a proof-of-concept for targeting KNTC1 therapeutically. Taken toghther, our study moves beyond descriptive oncology to mechanistically decipher a novel pathogenic circuit in BLCA. We redefine KNTC1 from a mitotic protein to a multi-functional oncogene that sits at the hub of a feed-forward loop with MYC, regulates a key signaling pathway, and drives chemoresistance. These insights not only deepen our understanding of BLCA biology but also open new avenues for combination therapies aimed at disrupting the KNTC1-E2F8-MYC axis for the treatment of advanced and chemoresistant BLCA.

## Conclusions

**Fig. 10:**
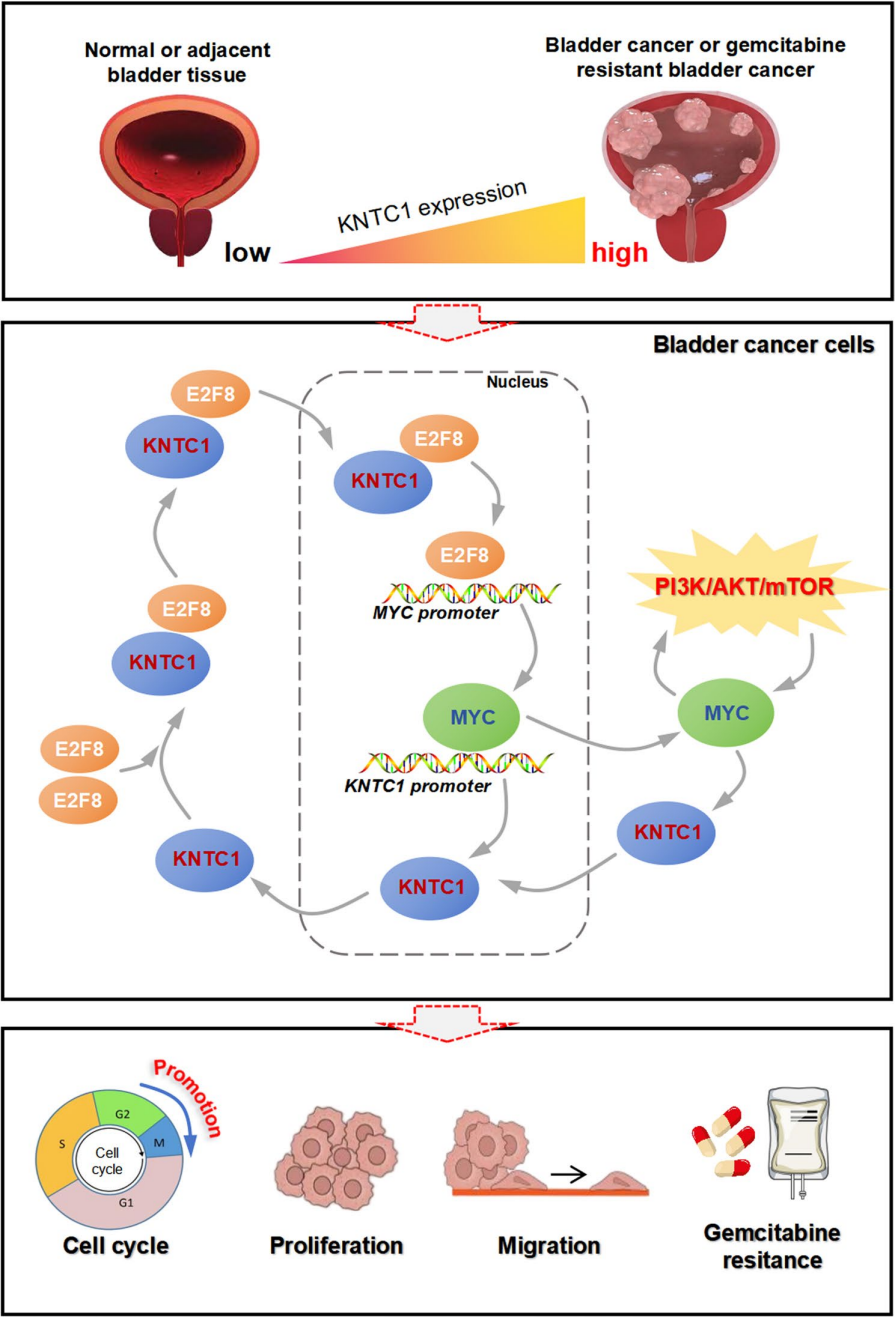
Schematic diagram showing KNTC1 promotes BLCA tumorigenesis and chemoresistance by orchestrating an E2F8-MYC positive feedback loop

This study concludes that KNTC1 drives BLCA progression and chemoresistance via the E2F8/MYC/PI3K-AKT axis, serving as both a prognostic biomarker and a promising therapeutic target (Fig. 10). Targeted inhibition of KNTC1 effectively suppresses tumor growth and metastasis, offering a novel strategic approach for BLCA treatment.

## Supplementary Information

Supplementary Material 1

Supplementary Material 2

Supplementary Material 3

Supplementary Material 4

Supplementary Material 5

Supplementary Material 6

Supplementary Material 7

Supplementary Material 8

Supplementary Material 9

Supplementary Material 10

Supplementary Material 11

Supplementary Material 12

Supplementary Material 13

Supplementary Material 14

Supplementary Material 15

Supplementary Material 16

Supplementary Material 17

Supplementary Material 18

Supplementary Material 19

Supplementary Material 20

Supplementary Material 21

Supplementary Material 22

Supplementary Material 23

Supplementary Material 24

## Data Availability

No datasets were generated or analysed during the current study.

## Abbreviations

BLCA: Bladder cCancer
ChIP: Chromatin Immunoprecipitation
Co-IP: Co-Immunoprecipitation
IF: Immunofuorescence
PAEs: poly(ß-amino ester)s
NSCLC: Non-small cell lung cancer
HCC: Hepatocellular carcinoma
ESCC: Esophageal squamous cell carcinoma
BLCA: Bladder cancer
OS: Overall survival
TCGA: The cancer genome atlas program
GEO: Gene expression omnibus

## References

[1] Siegel RL, Kratzer TB, Giaquinto AN, Sung H, Jemal A. Cancer statistics, 2025. CA Cancer J Clin. 2025;75(1):10–45. 10.3322/caac.21871. 39817679 10.3322/caac.21871 PMC11745215

[2] Lenis AT, Lec PM, Chamie K, Mshs MD. Bladder cancer: a review. JAMA. 2020;17(19):1980–91. 10.1001/jama.2020.17598. 10.1001/jama.2020.17598 33201207

[3] Compérat E, Amin MB, Cathomas R, Choudhury A, De Santis M, Kamat A, et al. Current best practice for bladder cancer: a narrative review of diagnostics and treatments. Lancet. 2022;400(10364):1712–21. 10.1016/S0140-6736(22)01188-6. 36174585 10.1016/S0140-6736(22)01188-6

[4] Babjuk M, Burger M, Capoun O, Cohen D, Compérat EM, Dominguez Escrig JL, et al. European association of urology guidelines on non-muscle-invasive bladder cancer (Ta, T1, and carcinoma in situ). Eur Urol. 2022;81(1):75–94. 10.1016/j.eururo.2021.08.010. 34511303 10.1016/j.eururo.2021.08.010

[5] Bellmunt J, de Wit R, Vaughn DJ, Fradet Y, Lee JL, Fong L, et al. Pembrolizumab as second-line therapy for advanced urothelial carcinoma. N Engl J Med. 2017;376(11):1015–26. 10.1056/NEJMoa1613683. 28212060 10.1056/NEJMoa1613683 PMC5635424

[6] Grivas P, Agarwal N, Pal S, Kalebasty AR, Sridhar SS, Smith J, et al. Avelumab first-line maintenance in locally advanced or metastatic urothelial carcinoma: applying clinical trial findings to clinical practice. Cancer Treat Rev. 2021;97:102187. 10.1016/j.ctrv.2021.102187. 33839438 10.1016/j.ctrv.2021.102187

[7] Naito S, Yokomizo A, Koga H. Mechanisms of drug resistance in chemotherapy for urogenital carcinoma. Int J Urol. 1999;6(9):427–39. 10.1046/j.1442-2042.1999.00088.x. 10510888 10.1046/j.1442-2042.1999.00088.x

[8] Wang M, Chen X, Tan P, Wang Y, Pan X, Lin T, et al. Acquired semi-squamatization during chemotherapy suggests differentiation as a therapeutic strategy for bladder cancer. Cancer Cell. 2022;12(9):1044-1059.e8. 10.1016/j.ccell.2022.08.010. 10.1016/j.ccell.2022.08.010 36099882

[9] Okobi TJ, Uhomoibhi TO, Akahara DE, Odoma VA, Sanusi IA, Okobi OE, et al. Immune checkpoint inhibitors as a treatment option for bladder cancer: current evidence. Cureus. 2023;15(6):e40031. 10.7759/cureus.40031. 37425564 10.7759/cureus.40031 PMC10323982

[10] Hushmandi K, Farahani N, Einollahi B, Salimimoghadam S, Alimohammadi M, Liang L, Liu L, Sethi G. Deciphering molecular pathways in urological cancers: A gateway to precision therapeutics. J Adv Res. 2025 Jun 12:S2090-1232(25)00395-9. doi: 10.7759/cureus.40031. Epub ahead of print. PMID: 40516913.10.1016/j.jare.2025.06.00940516913

[11] Bakhoum SF, Cantley LC. The multifaceted role of chromosomal instability in cancer and its microenvironment. Cell. 2018;174(6):1347–60. 10.1016/j.cell.2018.08.027. 30193109 10.1016/j.cell.2018.08.027 PMC6136429

[12] Guo G, Sun X, Chen C, Wu S, Huang P, Li Z, Dean M, Huang Y, Jia W, Zhou Q, Tang A, Yang Z, Li X, Song P, Zhao X, Ye R, Zhang S, Lin Z, Qi M, Wan S, Xie L, Fan F, Nickerson ML, Zou X, Hu X, Xing L, Lv Z, Mei H, Gao S, Liang C, Gao Z, Lu J, Yu Y, Liu C, Li L, Fang X, Jiang Z, Yang J, Li C, Zhao X, Chen J, Zhang F, Lai Y, Lin Z, Zhou F, Chen H, Chan HC, Tsang S, Theodorescu D, Li Y, Zhang X, Wang J, Yang H, Gui Y, Wang J, Cai Z. Whole-genome and whole-exome sequencing of bladder cancer identifies frequent alterations in genes involved in sister chromatid cohesion and segregation. Nat Genet. 2013;45(12):1459–63. 10.1038/ng.2798. Epub 2013 Oct 13. PMID: 24121792; PMCID: PMC7512009. 24121792 10.1038/ng.2798 PMC7512009

[13] Navarro AP, Cheeseman IM. Kinetochore assembly throughout the cell cycle. Semin Cell Dev Biol. 2021;117:62–74. 10.1016/j.semcdb.2021.03.008. 33753005 10.1016/j.semcdb.2021.03.008 PMC8384650

[14] Musacchio A, Salmon ED. The spindle-assembly checkpoint in space and time. Nat Rev Mol Cell Biol. 2007;8(5):379–93. 10.1038/nrm2163. 17426725 10.1038/nrm2163

[15] Scaërou F, Starr DA, Piano F, Papoulas O, Karess RE, Goldberg ML. The ZW10 and rough deal checkpoint proteins function together in a large, evolutionarily conserved complex targeted to the kinetochore. J Cell Sci. 2001;114(17):3103–14. 10.1242/jcs.114.17.3103. 11590237 10.1242/jcs.114.17.3103

[16] Chan GK, Jablonski SA, Starr DA, Goldberg ML, Yen TJ. Human Zw10 and ROD are mitotic checkpoint proteins that bind to kinetochores. Nat Cell Biol. 2000;2(12):944–7. 10.1038/35046598. 11146660 10.1038/35046598

[17] Liu R, Liu R, Guo Z, Ren J, Huang J, Luo Q, et al. Shrna-mediated knockdown of KNTC1 inhibits non-small-cell lung cancer through regulating PSMB8. Cell Death Dis. 2022;13(6):685. 10.1038/s41419-022-05140-w. 35933405 10.1038/s41419-022-05140-w PMC9357013

[18] Tong H, Liu X, Peng C, Shen B, Zhu Z. Silencing of KNTC1 inhibits hepatocellular carcinoma cells progression via suppressing PI3K/Akt pathway. Cell Signal. 2023;101:110498. 10.1016/j.cellsig.2022.110498. 36273753 10.1016/j.cellsig.2022.110498

[19] Liu CT, Min L, Wang YJ, Li P, Wu YD, Zhang ST. shRNA-mediated knockdown of KNTC1 suppresses cell viability and induces apoptosis in esophageal squamous cell carcinoma. Int J Oncol. 2019;54(3):1053–60. 10.3892/ijo.2019.4672. 30628654 10.3892/ijo.2019.4672

[20] Huang H, Fan X, Qiao Y, Yang M, Ji Z. Knockdown of KNTC1 inhibits the proliferation, migration and tumorigenesis of human bladder cancer cells and induces apoptosis. Crit Rev Eukaryot Gene Expr. 2021;31(1):49–60. 10.1615/CritRevEukaryotGeneExpr.2021037301. 33639055 10.1615/CritRevEukaryotGeneExpr.2021037301

[21] Chen S, Gao Y, Chen F, Wang TB. ANLN serves as an oncogene in bladder urothelial carcinoma via activating JNK signaling pathway. Urol Int. 2023;107(3):310–20. 10.1159/000524204. 35504258 10.1159/000524204

[22] Wang XX, Wu HY, Yang Y, Ma MM, Zhang YW, Huang HZ, et al. CCNB1 is involved in bladder cancer pathogenesis and silencing CCNB1 decelerates tumor growth and improves prognosis of bladder cancer. Exp Ther Med. 2023;26:382. 10.3892/etm.2023.12081. 37456156 10.3892/etm.2023.12081 PMC10347295

[23] Kim SK, Roh YG, Park K, Kang TH, Kim WJ, Lee JS, et al. Expression signature defined by FOXM1-CCNB1 activation predicts disease recurrence in non-muscle-invasive bladder cancer. Clin Cancer Res. 2014;20(15):3233–43. 10.1158/1078-0432.CCR-13-2761. 24714775 10.1158/1078-0432.CCR-13-2761

[24] Yang D, Ma Y, Zhao P, Ma J, He C. HMMR is a downstream target of FOXM1 in enhancing proliferation and partial epithelial-to-mesenchymal transition of bladder cancer cells. Exp Cell Res. 2021;408(15):112860. 10.1016/j.yexcr.2021.112860. 34624323 10.1016/j.yexcr.2021.112860

[25] Porta C, Paglino C, Mosca A. Targeting PI3K/Akt/mTOR signaling in cancer. Front Oncol. 2014;4(14):64. 10.3389/fonc.2014.00064. 24782981 10.3389/fonc.2014.00064 PMC3995050

[26] Sun J, Shi R, Zhao S, Li X, Lu S, Bu H, et al. E2F8, a direct target of miR-144, promotes papillary thyroid cancer progression via regulating cell cycle. J Exp Clin Cancer Res. 2017;36(7):40. 10.1186/s13046-017-0504-6. 28270228 10.1186/s13046-017-0504-6 PMC5341194

[27] Tian J, Lin Y, Yu J. E2F8 confers cisplatin resistance to ER + breast cancer cells via transcriptionally activating MASTL. Biomed Pharmacother. 2017;92:919–26. 10.1016/j.biopha.2017.05.118. 28605876 10.1016/j.biopha.2017.05.118

[28] Kim LK, Park SA, Eoh KJ, Heo TH, Kim YT, Kim HJ. E2F8 regulates the proliferation and invasion through epithelial-mesenchymal transition in cervical cancer. Int J Biol Sci. 2020;16(2):320–9. 10.7150/ijbs.37686. 31929759 10.7150/ijbs.37686 PMC6949145

[29] Liu LY, Tian L, Gao LH, Cui HJ, Li XM, Li YH. E2F8 facilitates malignant phenotypes of muscle-invasive bladder cancer via increasing MCM7 expression. Biochem Cell Biol. 2025;103:1–14. 10.1139/bcb-2024-0083. 39601318 10.1139/bcb-2024-0083

[30] Fragkoulis C, Ntoumas G, Glykas I, Papadopoulos G, Stathouros G, Kostopoulou A, et al. Expression of proto-oncogene c-Myc in patients with urinary bladder transitional cell carcinoma. Curr Urol. 2021;15(4):231–3. 10.1097/CU9.0000000000000053. 35069088 10.1097/CU9.0000000000000053 PMC8772624

[31] Schlack K, Boegemann M, Steinestel J, Schrader AJ, Krabbe LM. The safety and efficacy of gemcitabine for the treatment of bladder cancer. Expert Rev Anticancer Ther. 2016;16(3):255–71. 10.1586/14737140.2016.1143777. 26781169 10.1586/14737140.2016.1143777

[32] Han MA, Maisch P, Jung JH, Hwang JE, Narayan V, Cleves A, Hwang EC, Dahm P. Intravesical gemcitabine for non-muscle invasive bladder cancer. Cochrane Database Syst Rev. 2021;6(6):CD009294. 10.1002/14651858.CD009294.pub3. PMID: 34125951; PMCID: PMC8202966. 34125951 10.1002/14651858.CD009294.pub3 PMC8202966

[33] Xiong Y, Ju L, Yuan L, Chen L, Wang G, Xu H, et al. KNSTRN promotes tumorigenesis and gemcitabine resistance by activating AKT in bladder cancer. Oncogene. 2021;40(9):1595–608. 10.1038/s41388-020-01634-z. 33452459 10.1038/s41388-020-01634-z

[34] Rutz J, Maxeiner S, Juengel E, Chun FK, Tsaur I, Blaheta RA. Olive mill wastewater inhibits growth and proliferation of cisplatin- and gemcitabine-resistant bladder cancer cells in vitro by down-regulating the Akt/mTOR-signaling pathway. Nutrients. 2022;14(2):369. 10.3390/nu14020369. 35057550 10.3390/nu14020369 PMC8778865

[35] Mey V, Giovannetti E, De Braud F, Nannizzi S, Curigliano G, Verweij F, et al. In vitro synergistic cytotoxicity of gemcitabine and pemetrexed and pharmacogenetic evaluation of response to gemcitabine in bladder cancer patients. Br J Cancer. 2006;95(3):289–97. 10.1038/sj.bjc.6603242. (**Epub 2006 Jul 25. PMID: 16868547; PMCID: PMC2360654**). 16868547 10.1038/sj.bjc.6603242 PMC2360654

[36] Abida W, Milowsky MI, Ostrovnaya I, Gerst SR, Rosenberg JE, Voss MH, et al. Phase I study of everolimus in combination with gemcitabine and split-dose cisplatin in advanced urothelial carcinoma. Bladder Cancer. 2016;2(1):111–7 (**PMID: 27376132; PMCID: PMC4927849**). 27376132 10.3233/BLC-150038 PMC4927849

[37] Seo HK, Ahn KO, Jung NR, Shin JS, Park WS, Lee KH, et al. Antitumor activity of the c-Myc inhibitor KSI-3716 in gemcitabine-resistant bladder cancer. Oncotarget. 2014;5(30):326–37. 10.18632/oncotarget.1545. 24504118 10.18632/oncotarget.1545 PMC3964210

[38] Deng Z, Zhou F, Li M, Jin W, Yu J, Wang G, et al. DLGAP5 enhances bladder cancer chemoresistance by regulating glycolysis through MYC stabilization. Theranostics. 2025;15(20):2375–92. 10.7150/thno.102730. 39990228 10.7150/thno.102730 PMC11840727

[39] Lin CP, Liu JD, Chow JM, Liu CR, Liu HE. Small-molecule c-Myc inhibitor, 10058-F4, inhibits proliferation, downregulates human telomerase reverse transcriptase and enhances chemosensitivity in human hepatocellular carcinoma cells. Anticancer Drugs. 2007;18(2):161–70. 10.1097/CAD.0b013e3280109424. 17159602 10.1097/CAD.0b013e3280109424

[40] Zhang M, Fan HY, Li SC. Inhibition of c-Myc by 10058-F4 induces growth arrest and chemosensitivity in pancreatic ductal adenocarcinoma. Biomed Pharmacother. 2015;73:123–8. 10.1016/j.biopha.2015.05.019. 26211592 10.1016/j.biopha.2015.05.019

[41] Jere D, Xu CX, Arote R, Yun CH, Cho MH, Cho CS. Poly(beta-amino ester) as a carrier for si/shRNA delivery in lung cancer cells. Biomaterials. 2008;29(16):2535–47. 10.1016/j.biomaterials.2008.02.018. 18316120 10.1016/j.biomaterials.2008.02.018

[42] Nabar N, Dacoba TG, Covarrubias G, Romero-Cruz D, Hammond PT. Electrostatic adsorption of polyanions onto lipid nanoparticles controls uptake, trafficking, and transfection of RNA and DNA therapies. Proc Natl Acad Sci U S A. 2024;121(12):e2307809121. 10.1073/pnas.2307809121. 38437543 10.1073/pnas.2307809121 PMC10945854

[43] Giraud L, Viricel W, Leblond J, Giasson S. Single stranded siRNA complexation through non-electrostatic interactions. Biomaterials. 2017;113:230–42. 10.1016/j.biomaterials.2016.10.035. 27825070 10.1016/j.biomaterials.2016.10.035

[44] Du A, Li S, Zhou Y, Disoma C, Liao Y, Zhang Y, et al. M6A-mediated upregulation of circMDK promotes tumorigenesis and acts as a nanotherapeutic target in hepatocellular carcinoma. Mol Cancer. 2022;21(6):109. 10.1186/s12943-022-01575-z. 35524319 10.1186/s12943-022-01575-z PMC9074191

[45] Liu S, Gao Y, Zhou D, Zeng M, Alshehri F, Newland B, et al. Highly branched poly(ß-amino ester) delivery of minicircle DNA for transfection of neurodegenerative disease related cells. Nat Commun. 2019;10(1):3307. 10.1038/s41467-019-11190-0. 31341171 10.1038/s41467-019-11190-0 PMC6656726

[46] Borhaninia M, Zahiri M, Abnous K, Taghdisi SM, Ramezani M, Alibolandi M. Self-targeted hyaluronic acid-b-poly (ß-amino ester) pH-switchable polymersome for guided doxorubicin delivery to metastatic breast cancer. Int J Biol Macromol. 2023;248:125882. 10.1016/j.ijbiomac.2023.125882. 37473882 10.1016/j.ijbiomac.2023.125882

