# Supplementary material for: KNTC1 initiates a KNTC1/E2F8/MYC positive feedback loop to facilitate tumorigenesis and enhance chemoresistance in bladder cancer
Source: J Exp Clin Cancer Res. 2026 Feb 4;45:38. doi: 10.1186/s13046-026-03651-4 (PMC12879452; doi:10.1186/s13046-026-03651-4)

Fig. S1

A

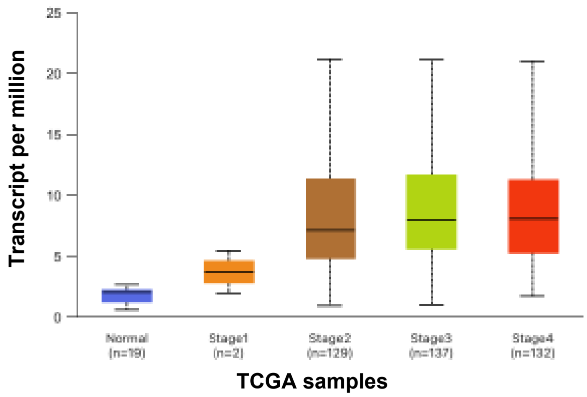

Comparison	Statistical significance
Normal-vs-Stage1	3.073800E-01
Normal-vs-Stage2	<1E-12
Normal-vs-Stage3	<1E-12
Normal-vs-Stage4	1.62447832963153E-12
Stage1-vs-Stage2	2.403600E-01
Stage1-vs-Stage3	1.794590E-01
Stage1-vs-Stage4	1.263570E-01
Stage2-vs-Stage3	8.775800E-01
Stage2-vs-Stage4	8.238200E-01
Stage3-vs-Stage4	6.731600E-01

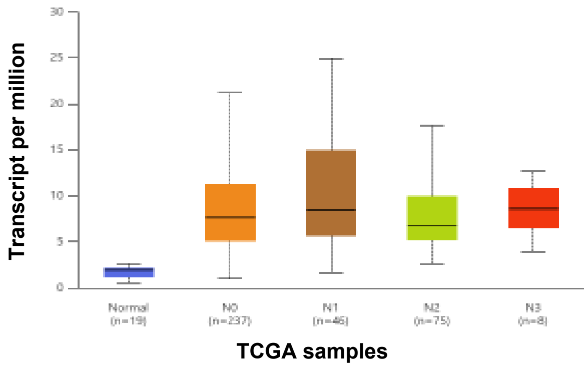

Comparison	Statistical significance
Normal-vs-N0	1.62447832963153E-12
Normal-vs-N1	1.49890100331618E-11
Normal-vs-N2	2.1094237467878E-15
Normal-vs-N3	5.980800E-04
N0-vs-N1	2.499200E-01
N0-vs-N2	6.796000E-01
N0-vs-N3	7.848800E-01
N1-vs-N2	1.659610E-01
N1-vs-N3	4.570600E-01
N2-vs-N3	8.832800E-01

B

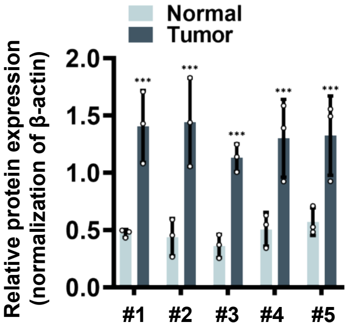

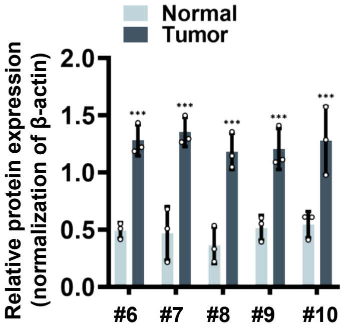

Supplement: Supplementary file 1 — Supplementary Material 1 [file 13046_2026_3651_MOESM1_ESM.pdf]
