# Supplementary figures and images for: KNTC1 initiates a KNTC1/E2F8/MYC positive feedback loop to facilitate tumorigenesis and enhance chemoresistance in bladder cancer
Source: J Exp Clin Cancer Res. 2026 Feb 4;45:38. doi: 10.1186/s13046-026-03651-4 (PMC12879452; doi:10.1186/s13046-026-03651-4)

Fig. S2

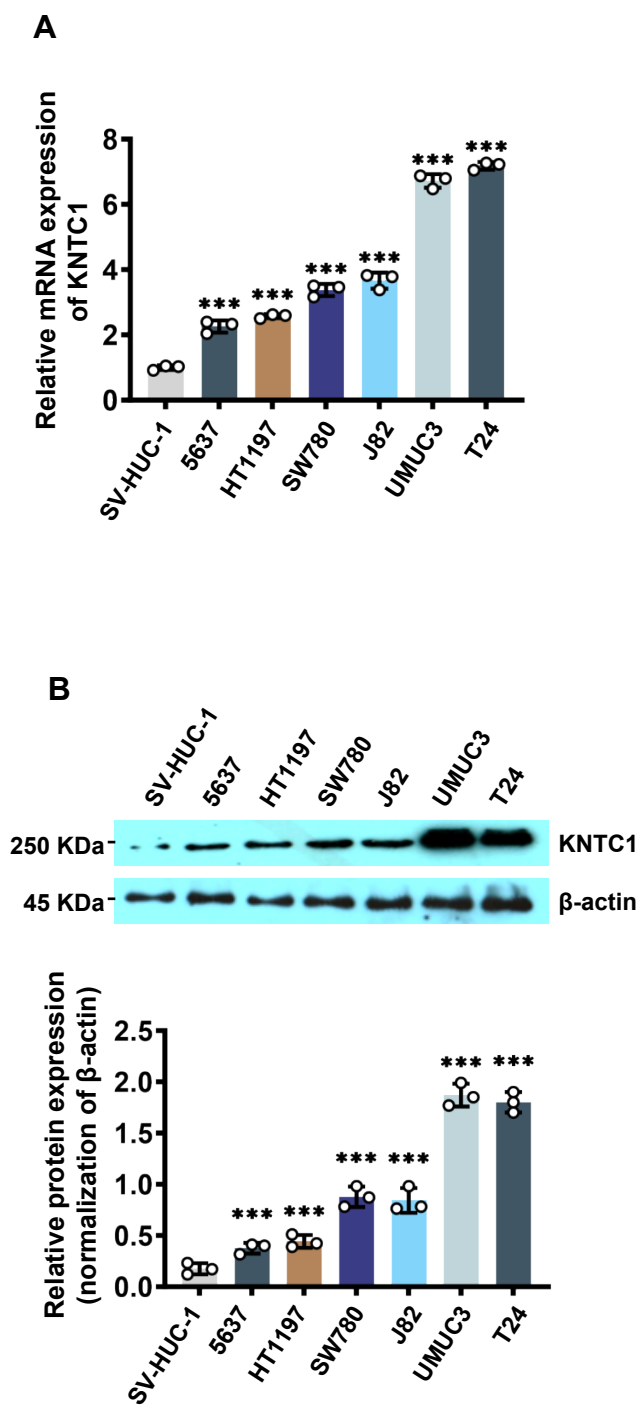

Supplement: Supplementary file 2 — Supplementary Material 2 [file 13046_2026_3651_MOESM2_ESM.pdf]

Fig. S3

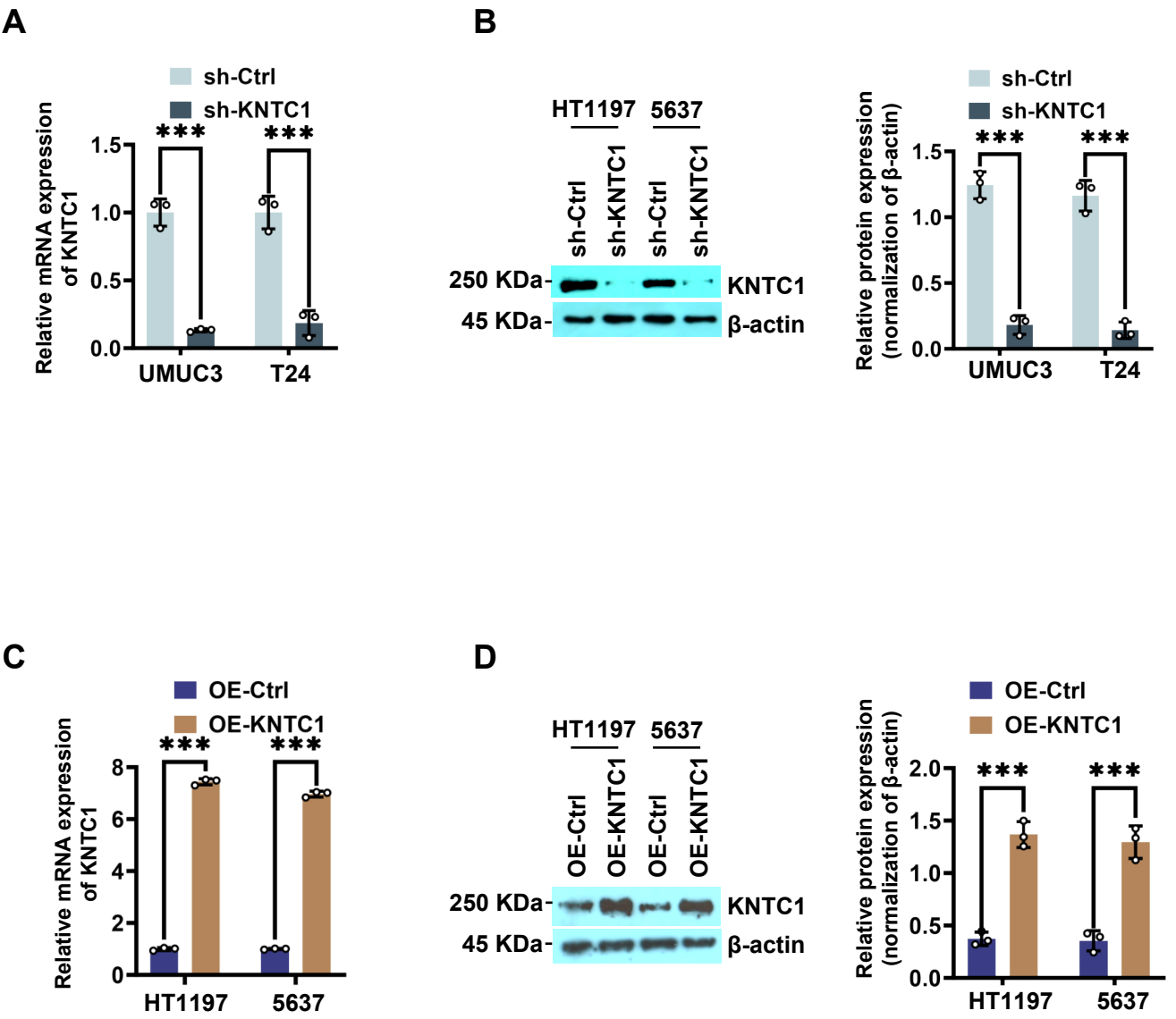

Supplement: Supplementary file 3 — Supplementary Material 3 [file 13046_2026_3651_MOESM3_ESM.pdf]

Fig. S4

*KNTC1* vs *CCNB1*

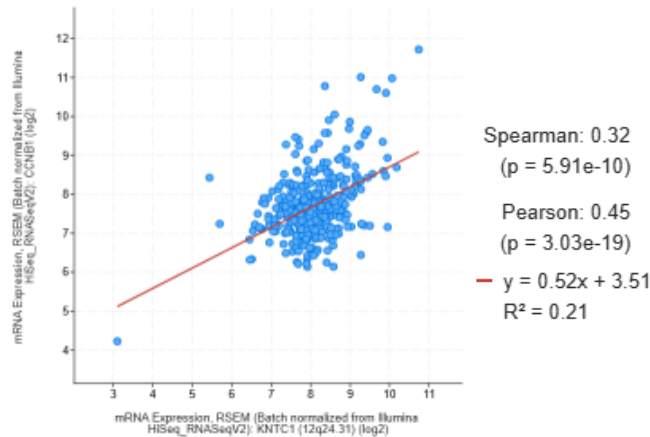

*KNTC1* vs *CDK1*

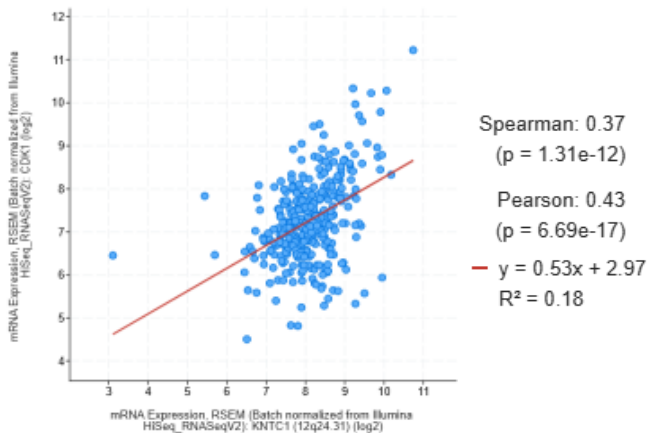

*KNTC1* vs *CCNE1*

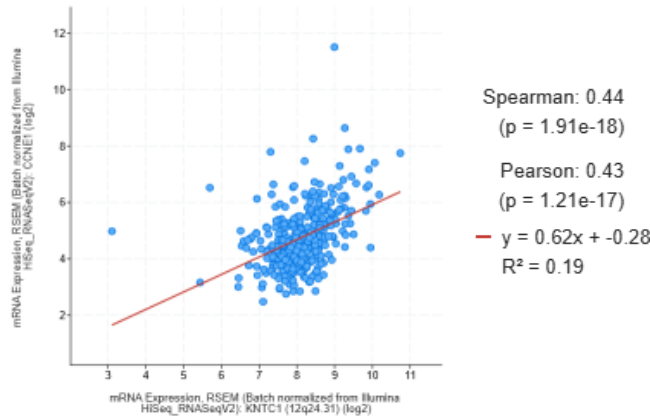

*KNTC1* vs *CDK2*

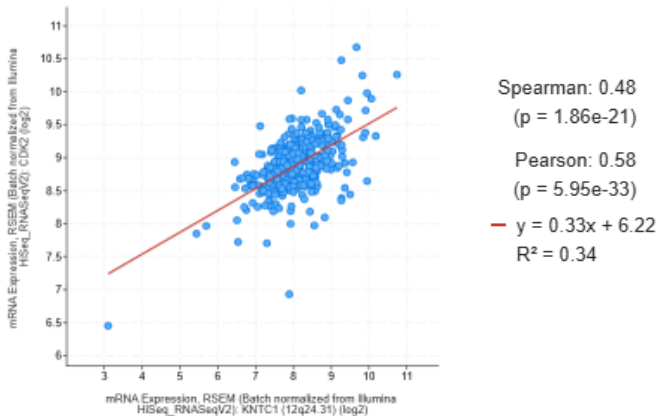

*KNTC1* vs *CCNA1*

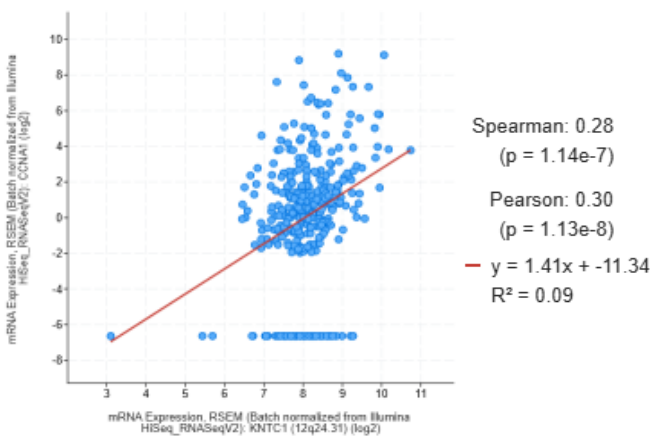

Supplement: Supplementary file 4 — Supplementary Material 4 [file 13046_2026_3651_MOESM4_ESM.pdf]

Fig. S5

A

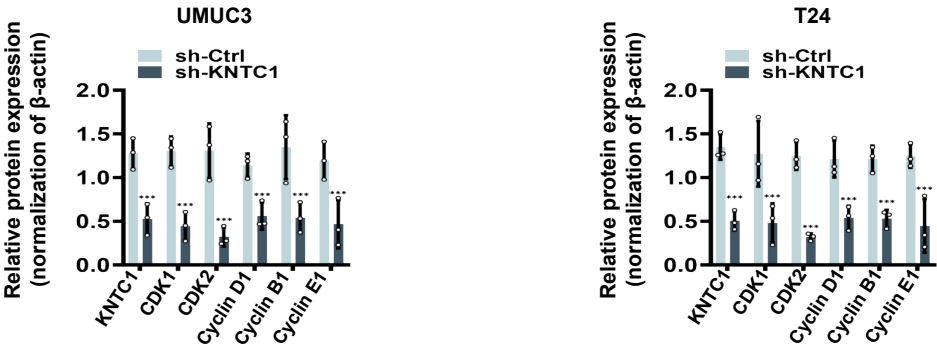

B

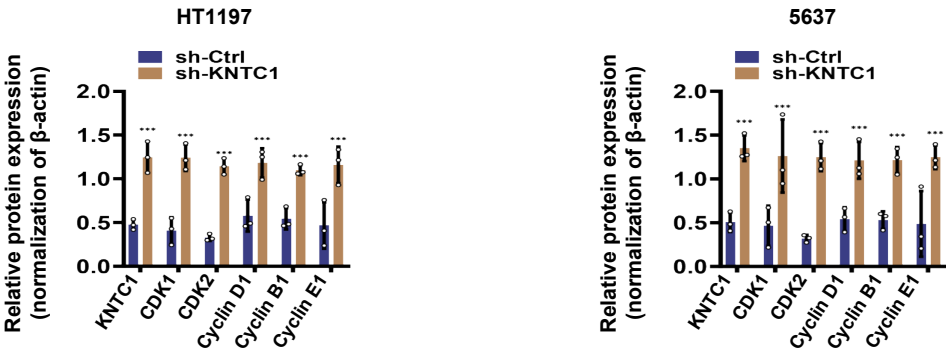

Supplement: Supplementary file 5 — Supplementary Material 5 [file 13046_2026_3651_MOESM5_ESM.pdf]

Fig. S6

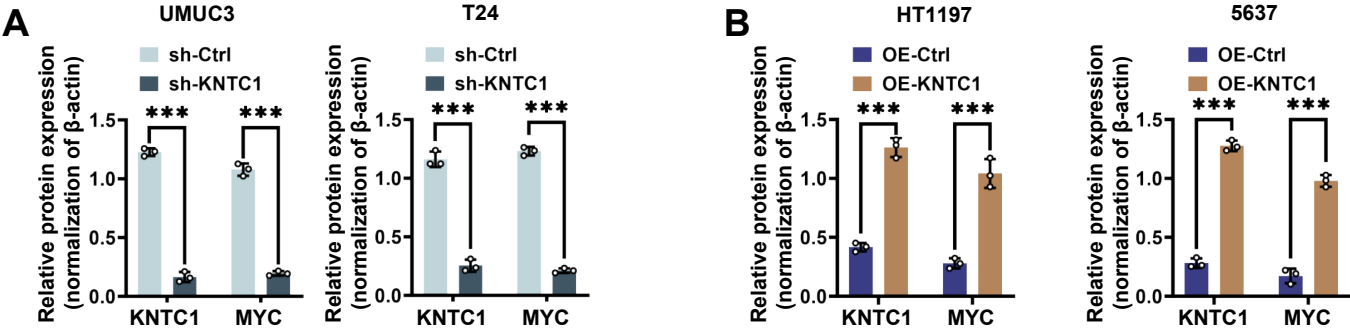

Supplement: Supplementary file 6 — Supplementary Material 6 [file 13046_2026_3651_MOESM6_ESM.pdf]

Fig. S7

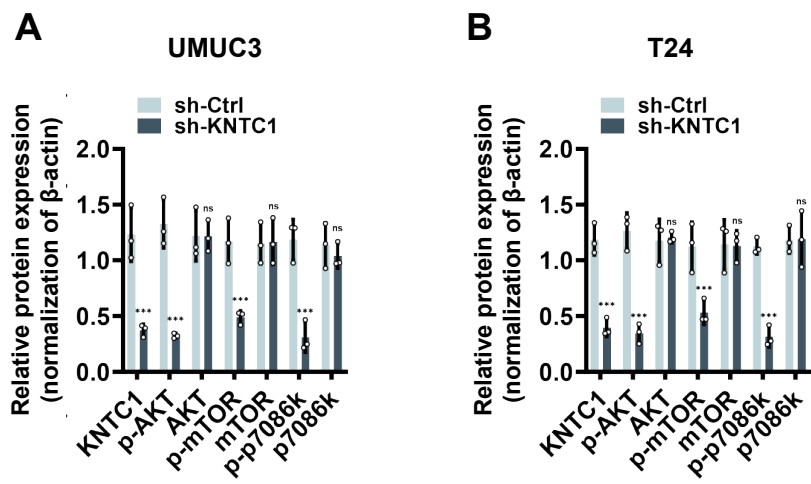

Supplement: Supplementary file 7 — Supplementary Material 7 [file 13046_2026_3651_MOESM7_ESM.pdf]

Fig. S8

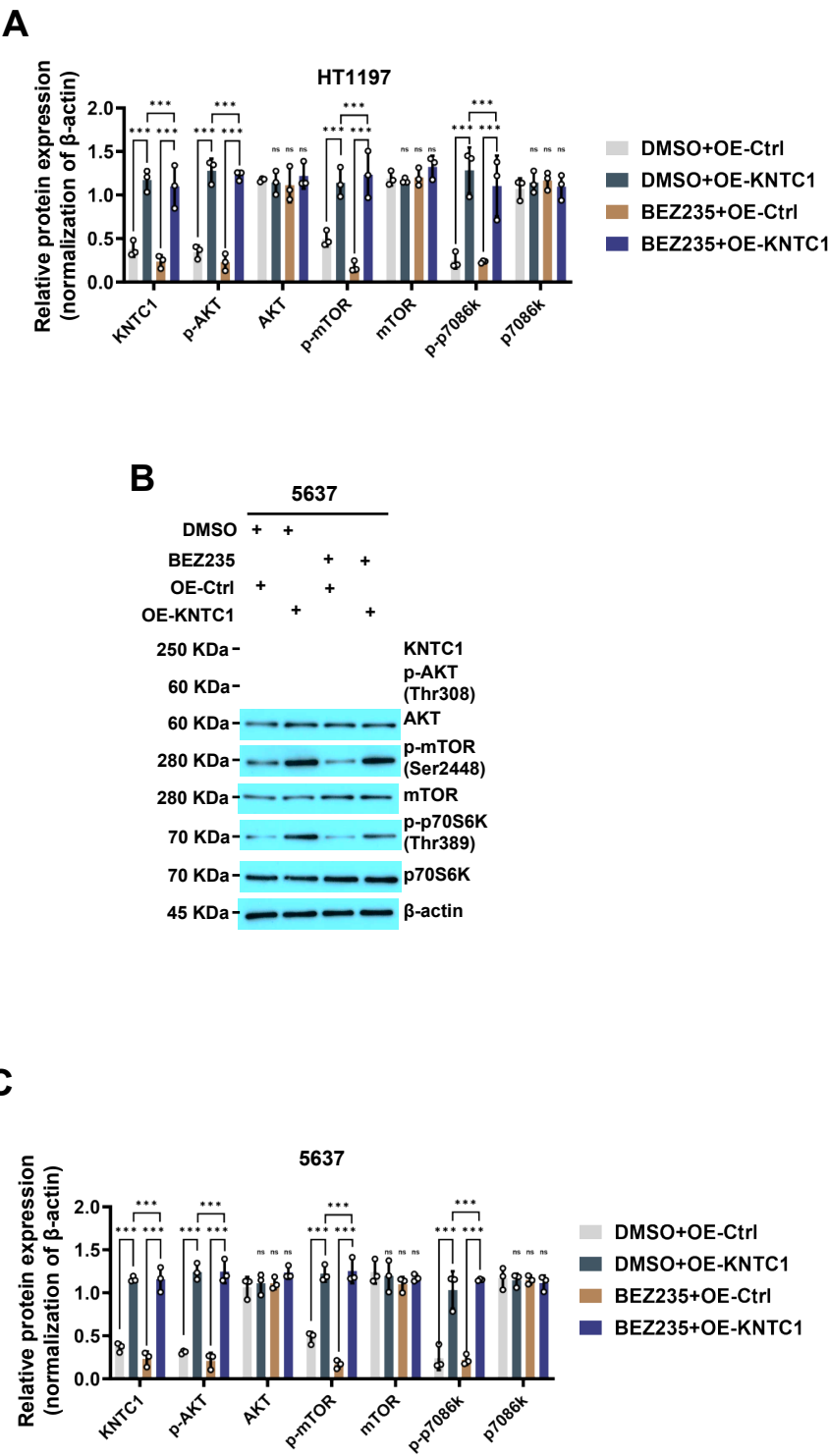

Supplement: Supplementary file 8 — Supplementary Material 8 [file 13046_2026_3651_MOESM8_ESM.pdf]

Fig. S9

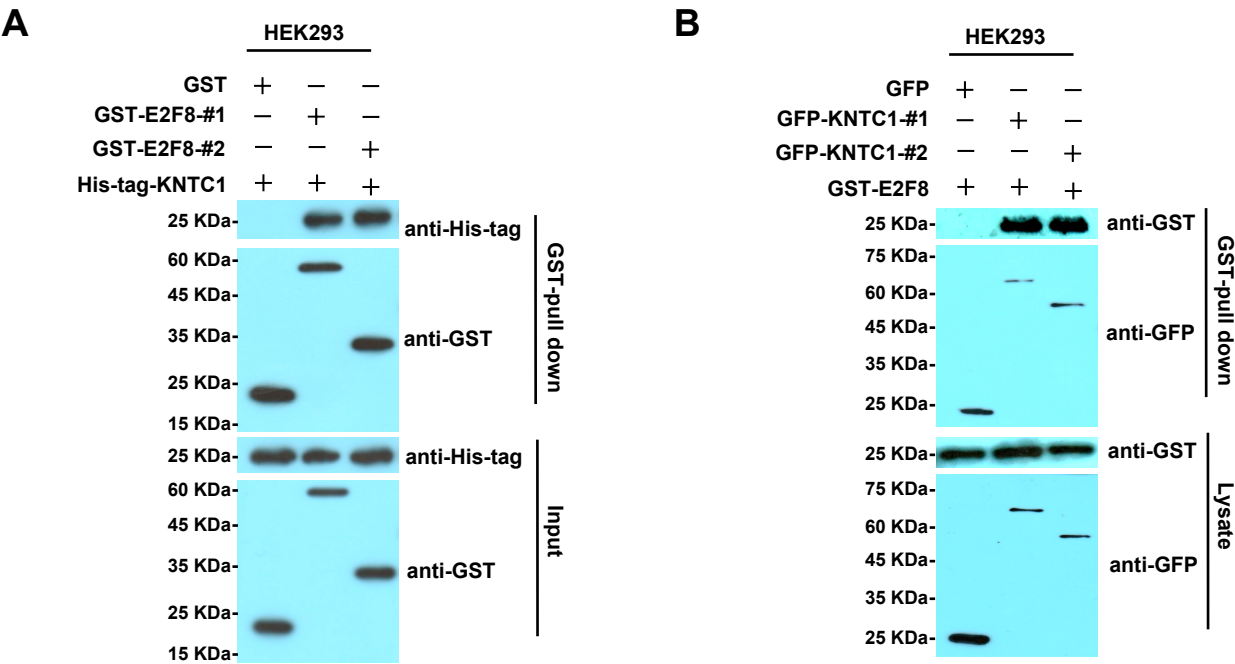

Supplement: Supplementary file 9 — Supplementary Material 9 [file 13046_2026_3651_MOESM9_ESM.pdf]

Fig. S10

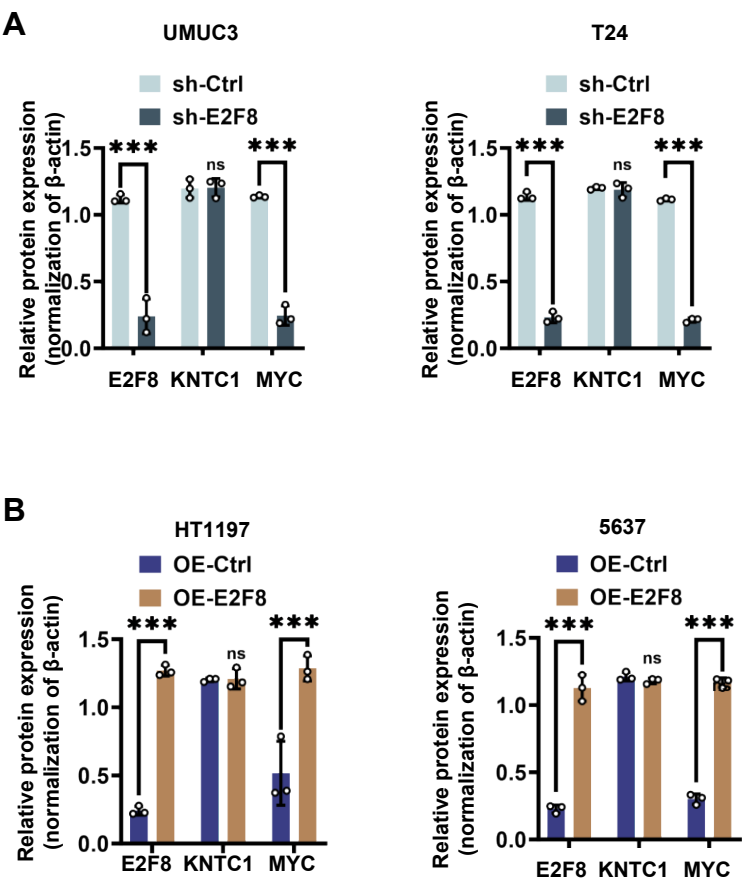

Supplement: Supplementary file 10 — Supplementary Material 10 [file 13046_2026_3651_MOESM10_ESM.pdf]

Fig. S11

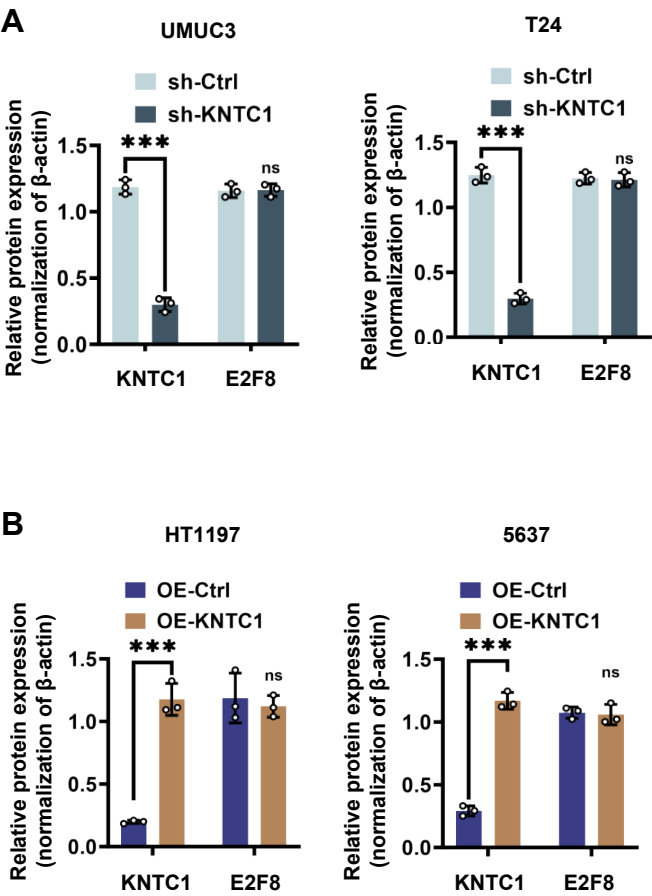

Supplement: Supplementary file 11 — Supplementary Material 11 [file 13046_2026_3651_MOESM11_ESM.pdf]

Fig. S12

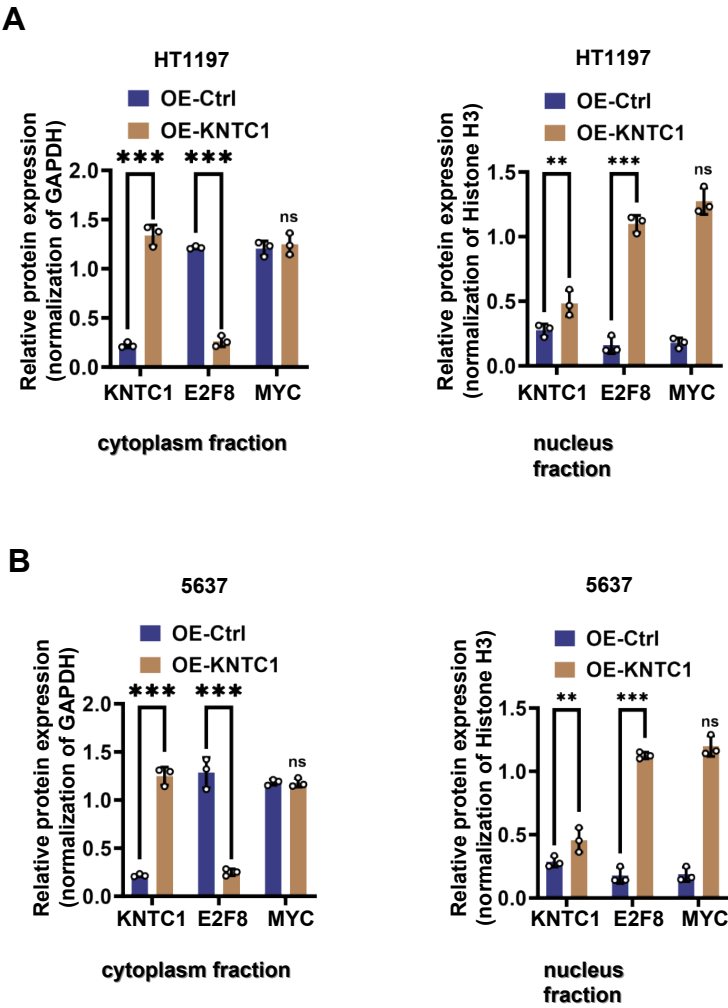

Supplement: Supplementary file 12 — Supplementary Material 12 [file 13046_2026_3651_MOESM12_ESM.pdf]

Fig. S13

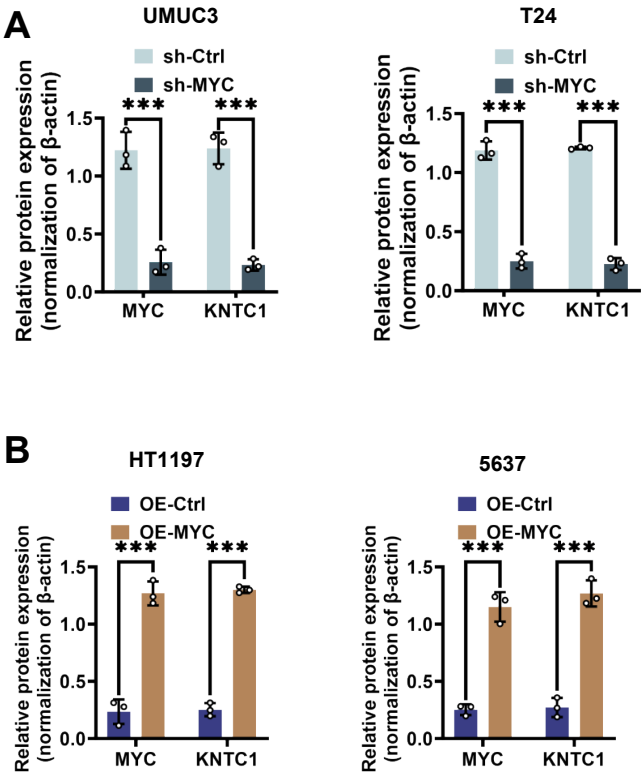

Supplement: Supplementary file 13 — Supplementary Material 13 [file 13046_2026_3651_MOESM13_ESM.pdf]

Fig. S14

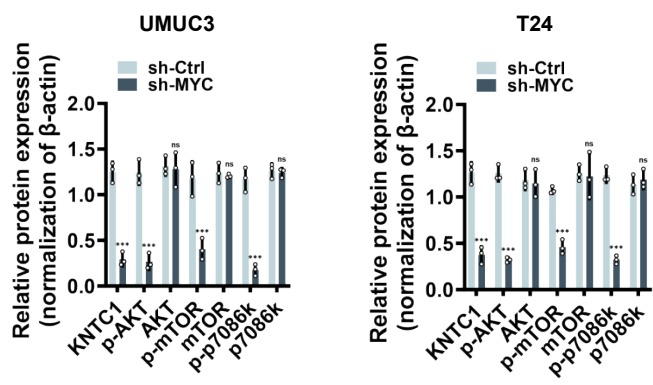

Supplement: Supplementary file 14 — Supplementary Material 14 [file 13046_2026_3651_MOESM14_ESM.pdf]

Fig. S15

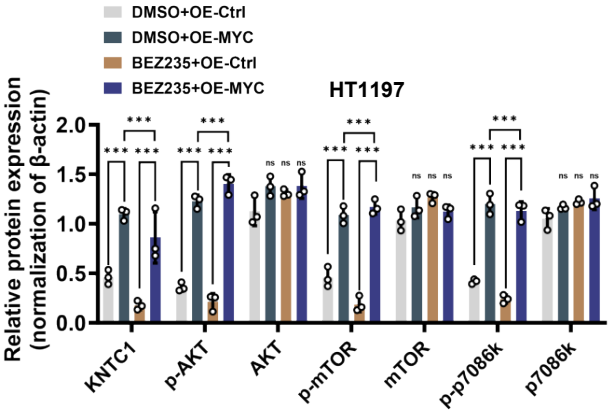

Supplement: Supplementary file 15 — Supplementary Material 15 [file 13046_2026_3651_MOESM15_ESM.pdf]

Fig. S16

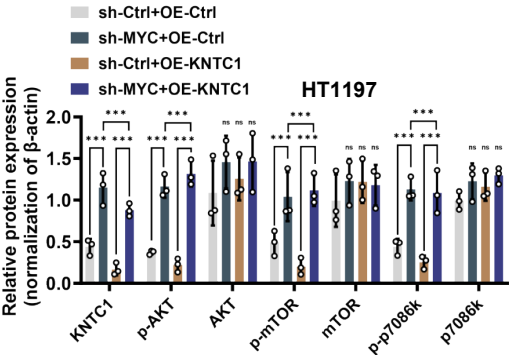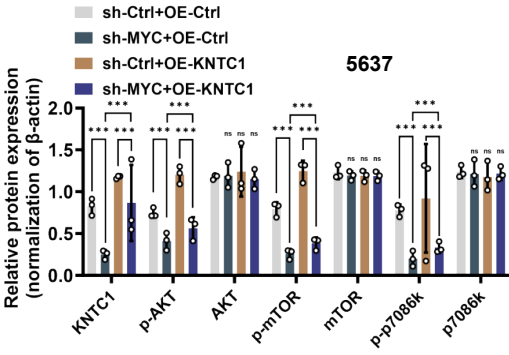

Supplement: Supplementary file 16 — Supplementary Material 16 [file 13046_2026_3651_MOESM16_ESM.pdf]

Fig. S17

A

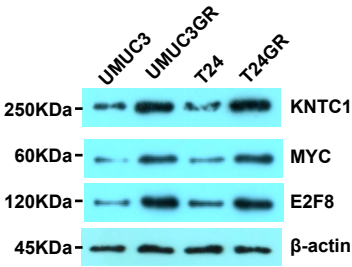

B

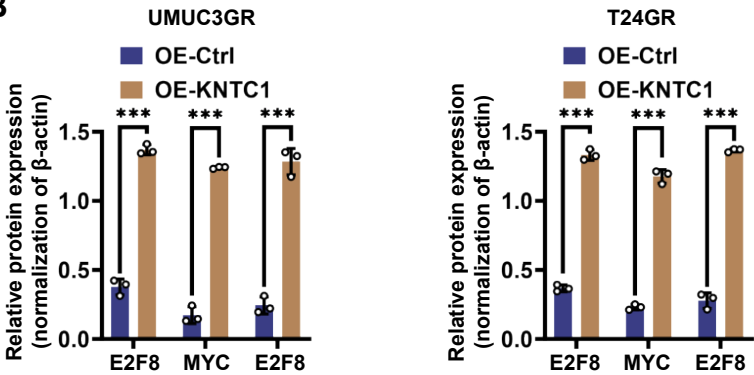

Supplement: Supplementary file 17 — Supplementary Material 17 [file 13046_2026_3651_MOESM17_ESM.pdf]

Fig. S18

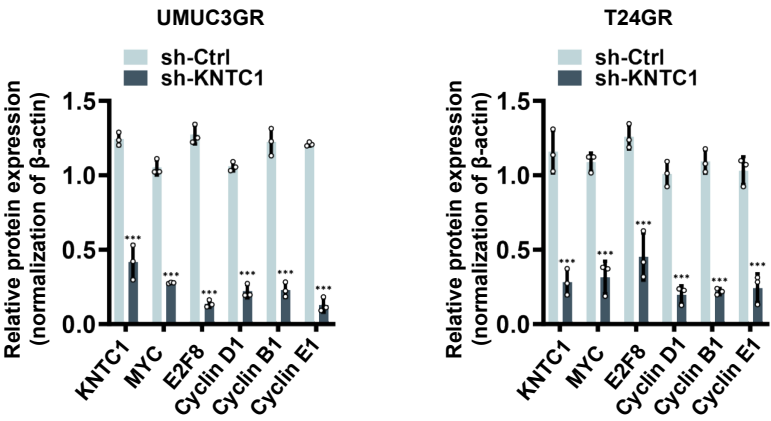

Supplement: Supplementary file 18 — Supplementary Material 18 [file 13046_2026_3651_MOESM18_ESM.pdf]
