# Supplementary material for: KNTC1 initiates a KNTC1/E2F8/MYC positive feedback loop to facilitate tumorigenesis and enhance chemoresistance in bladder cancer
Source: J Exp Clin Cancer Res. 2026 Feb 4;45:38. doi: 10.1186/s13046-026-03651-4 (PMC12879452; doi:10.1186/s13046-026-03651-4)
Supplement: Supplementary file 19 — Supplementary Material 19 [file 13046_2026_3651_MOESM19_ESM.docx]

**Supplementary figure legends:**

**Fig. S1 High expression of KNTC1 is associated with malignant progression in BLCA.**

**A** The mRNA level of *KNTC1* in BLCA tissues from different cancer stages and metastasis status. **B** The quantitative analysis of the protein levels of KNTC1 in our collected BLCA tissues and paired adjacent normal tissues. ns, no significance; * *P* < 0.05; ** *P* < 0.01; *** *P* < 0.001.

**Fig. S2 KNTC1 is highly expressed in BLCA cells.**

**A and B** The expression levels of KNTC1 in BLCA cell lines (5637, HT1197, SW780, J82, UMUC3, T24) and the human immortalized urothelial cell line SV-HUC-1 were analyzed by RT-qPCR and western blotting. ns, no significance; * *P* < 0.05; ** *P* < 0.01; *** *P* < 0.001.

**Fig. S3 Analysis of efficiency of sh-KNTC1 in BLCA cells.**

The knockdown and overexpression efficiency of KNTC1 in BLCA cell lines was verified by RT-qPCR and western blotting. ns, no significance; * *P* < 0.05; ** *P* < 0.01; *** *P* < 0.001.

**Fig. S4 The correlation between KNTC1 and cell cycle-related factors in BLCA.**

The correlation between *KNTC1* mRNA and cell cycle-related factors mRNA expression was assessed in BLCA patient samples from the TCGA-BLCA cohort.

**Fig. S5  Quantification of western blotting data in Figure 2G.**

Quantification of cell cycle-associated protein levels in BLCA cells. ns, no significance; * *P* < 0.05; ** *P* < 0.01; *** *P* < 0.001.

**Fig. S6  Quantification of western blotting data in Figures 3K and 3M.**

Quantification of KNTC1 and MYC proteins changes in BLCA cells. ns, no significance; * *P* < 0.05; ** *P* < 0.01; *** *P* < 0.001.

**Fig. S7  Quantification of western blotting data in Figure 3N.**

Western blotting quantitative analysis of p-AKT, p-mTOR, and p-p70S6K protein levels after KNTC1 knockdown. ns, no significance; * *P* < 0.05; ** *P* < 0.01; *** *P* < 0.001.

**Fig. S8 The phosphorylation status of the PI3K/AKT/mTOR pathway after OE-KNTC1 or treatment with the PI3K/AKT/mTOR inhibitor BEZ235 was examined by western blotting analysis and quantitative analysis.**

**A** Analysis quantifying the relative protein expression of KNTC1, p-AKT, AKT, p-mTOR, mTOR, p-p70S6K, and p70S6K in HT1197 cells. **B and** **C** Western blotting showed that after **treatment with BEZ235** or overexpression of KNTC1, the protein levels of KNTC1, p-AKT, AKT, p-mTOR, mTOR, p-p70S6K, and p70S6K were detected in 5637 cells. ns, no significance; * *P* < 0.05; ** *P* < 0.01; *** *P* < 0.001.

**Fig. S9 GST-pull down assay analyzed the interaction between E2F8 and KNTC1 in HEK293 cells.**

GST or GST fusion proteins were incubated with the indicated proteins and the bound proteins were analyzed by SDS-PAGE. GFP-tagged proteins and GST-tagged proteins were detected by western blotting.

**Fig. S10  Quantification of western blotting data in Figures 5C and 5D.**

**A** Analysis quantifying the relative protein expression of KNTC1, E2F8 and MYC after knockdown E2F8 in BLCA cells. **B** Protein-level quantification of KNTC1, E2F8 and MYC after overexpressed E2F8 in BLCA cells. ns, no significance; * *P* < 0.05; ** *P* < 0.01; *** *P* < 0.001.

**Fig. S11 Quantification of western blotting data in Figures 5H and 5J.**

**A** Quantified protein expression analysis of KNTC1 and E2F8 in sh-Ctrl/sh-KNTC1 BLCA cells. **B** Quantitative evaluation of KNTC1 and E2F8 protein levels in OE-Ctrl/OE-KNTC1 BLCA cells. ns, no significance; * *P* < 0.05; ** *P* < 0.01; *** *P* < 0.001.

**Fig. S12 Quantification of western blotting data in Figure 5K.**

**A and B** Quantitative profiling of KNTC1, E2F8 and MYC in the BLCA cell’s nuclear and cytoplasm after OE-KNTC1. ns, no significance; * *P* < 0.05; ** *P* < 0.01; *** *P* < 0.001.

**Fig. S13 Quantification of western blotting data in Figures 7B and 7D.**

**A and B** Quantitative results demonstrating the protein expression of KNTC1, MYC in BLCA cells after sh-KNTC1 or OE-KNTC1. ns, no significance; * *P* < 0.05; ** *P* < 0.01; *** *P* < 0.001.

**Fig. S14 Quantification of western blotting data in Figure 7I .**

**A and B** Quantitative results demonstrating the protein expression of KNTC1, MYC, p-AKT, AKT, p-mTOR, mTOR, p-p70S6K, and p70S6K in sh-MYC BLCA cells. ns, no significance; * *P* < 0.05; ** *P* < 0.01; *** *P* < 0.001.

**Fig. S15 Quantification of western blotting data in Figure 7J.**

OE-MYC reversed the inhibitory effect of the PI3K/AKT/mTOR inhibitor BEZ235 on the PI3K/AKT/mTOR pathway ns, no significance; * *P* < 0.05; ** *P* < 0.01; *** *P* < 0.001.

**Fig. S16 Quantification of western blotting data in Figure 7K.**

**A and B** Overexpression of KNTC1 rescued the suppression of PI3K/AKT/mTOR phosphorylation induced by sh-MYC. ns, no significance; * *P* < 0.05; ** *P* < 0.01; *** *P* < 0.001.

**Fig. S17 KNTC1, MYC and E2F8 are overexpressed in UMUC3/GR and T24/GR cells.**

**A and B** Western blotting and quantitative analysis of the expression of KNTC1, MYC and E2F8 are overexpressed in UMUC3/GR and T24/GR cells. ns, no significance; * *P* < 0.05; ** *P* < 0.01; *** *P* < 0.001.

**Fig. S18 Quantification of western blotting data in Figure 8F. ns, no significance; * *P* < 0.05; ** *P* < 0.01; *** *P* < 0.001.**
