# Supplementary material for: KNTC1 initiates a KNTC1/E2F8/MYC positive feedback loop to facilitate tumorigenesis and enhance chemoresistance in bladder cancer
Source: J Exp Clin Cancer Res. 2026 Feb 4;45:38. doi: 10.1186/s13046-026-03651-4 (PMC12879452; doi:10.1186/s13046-026-03651-4)
Supplement: Supplementary file 24 — Supplementary Material 24 [file 13046_2026_3651_MOESM24_ESM.docx]

**Supplementary Materials and Methods**

**Lentivirus infection**

Lentiviral constructs for KNTC1 manipulation were acquired from Fenghbio (Hunan, China). Two experimental configurations were generated: KNTC1-overexpressing vector (pLV-CMV-KNTC1-EF1-ZsGreen1-T2A-Puro), MYC-overexpressing vector (pLV-CMV-MYC-EF1-ZsGreen1-T2A-Puro), E2F8-overexpressing vector (pLV-CMV-E2F8-EF1-ZsGreen1-T2A-Puro) and empty vector control (pLV-CMV-EF1-ZsGreen1-T2A-Puro). ShRNA-targeting knocking down of KNTC1 (pLV-CMV-sh-KNTC1-EF1-ZsGreen1-T2A-Puro), ShRNA-targeting knocking down of MYC (pLV-CMV-sh-MYC-EF1-ZsGreen1-T2A-Puro), ShRNA-targeting knocking down of E2F8 (pLV-CMV-sh-E2F8-EF1-ZsGreen1-T2A-Puro) and scrambled shRNA control (pLV-CMV-EF1-ZsGreen1-T2A-Puro). The shRNA sequences were listed in Supplementary Table 1. Lentiviruses were employed to transduce BLCA cells with polybrene added (8 µg/mL; Sigma-Aldrich). Following infection, cells were selected with puromycin (2 µg/mL; Merck) for a period of two weeks. The efficiency of the infection was then assessed through western blot and RT-qPCR analysis.

**Gemcitabine-resistant cell lines**

The gemcitabine-resistant BLCA cell lines UMUC3/GR and T24/GR were established and characterized by the Suzhou Haixing Biological Technology Co., Ltd. (Suzhou, China).

**Reverse transcription quantitative PCR (RT-qPCR)**

Post collection, tissues and cells were immediately lysed in BeyoZol (Beyotime, cat. no. R0011). Total RNA was purified using a silica-based column purification kit (Servicebio), cDNA was generated from 1 μg RNA samples through reverse transcription with M-MLV enzyme (Tsingke) and oligo(dT) primers. Quantitative real-time PCR (RT-qPCR) assays were carried out on a LightCycler 480 system (Roche) employing SsoAdvanced™ Universal SYBR® Green Supermix (Bio-Rad, USA). The PCR procedure included an initial 5 min denaturation at 95°C, then 30 cycles of 94°C for 45 s, 60°C for 30 s, and 72°C for 5 min. All primers sequences were supplied by Sangon Biotech (Shanghai, China). Relative gene expression was measured employing the 2^-ΔΔCt^ quantification approach, normalized to β-actin (*ACTB*) as the endogenous control. Supplementary Table 2 contains the complete set of primer sequences used in this study.

**Western blotting assay**

Western blotting assay was conducted following established protocols as previously described [1]. Protein lysates were extracted, separated by SDS-PAGE, transferred onto NC membranes, and probed using standard procedures with primary antibodies. The dilution rate of primary antibodies is 1: 1000, the dilution rates of secondary antibodies is 1:10000. We used 20 μg of total protein for western blotting analysis indicated. The antibodies were listed in Supplementary Table 3.

**Chromatin immunoprecipitation (ChIP)**

5×10⁵ cells underwent formaldehyde fixation (1% final concentration) to induce protein-DNA crosslinking, with glycine added to quench the reaction. Cells were lysed and chromatin was sonicated to generate 500-1000 bp DNA fragments. Immunoprecipitation was conducted by incubating equal chromatin aliquots overnight at 4°C with 1 μg of anti-KNTC1 antibody or control anti-IgG. Protein-DNA complexes were then de-crosslinked to liberate bound DNA fragments. Purified DNA was quantified by real-time quantitative PCR (RT-qPCR) using primers specified in Supplementary Table 4.

**Proximity ligation assays (PLA)**

PLA was conducted in strict adherence to the manufacturer's established protocol, as outlined in prior work [2]. The Duolink In Situ PLA Probe Anti-Rabbit MINUS (Cat. DUO92002, Sigma-Aldrich, St. Louis, MO, USA) was employed to target KNTC1 (using Rabbit anti-KNTC1 antibody, Cat. 24694-1-AP, Proteintech), while the Duolink In Situ PLA Probe Anti-Mouse PLUS (Cat. DUO92004, Sigma-Aldrich) was utilized for E2F8 detection (using Mouse anti-E2F8 antibody, Cat. BF9529, Affinity Biotech). In brief, cells were cultured on coverslips within 12-well plates and subjected to the indicated shRNA treatment. Following this, cell fixation was performed using methanol for a duration of 5 minutes. After blocking with a suitable solution, the cells were incubated with specific primary antibody pairs at 37℃ for 1 hour. Subsequently, PLA probes, comprising secondary antibodies conjugated for ligation, were applied and incubated at 37°C for 30 minutes. This was followed by an amplification step carried out at 37°C for 100 minutes. Afterwards, nuclei were counterstained with DAPI (Cat. DUO82040, Sigma-Aldrich) for 10 minutes. For every experimental sample, a minimum of 100 cells were evaluated. Image analysis and quantification were performed utilizing ImageJ software (Version 1.52, NIH, Bethesda, MD, USA). PLA signal dots were quantified from 8-bit images via the "Analyze Particles" function, and cell counting was conducted using the cell counter plugin.

**Glutathione S-Transferase (GST) pull down analysis**

To perform GST pull-down assays with cellular lysates, HEK293 or UMUC3 cells were harvested and lysed in P150 buffer (50 mM Tris-HCl, pH 7.5, 150 mM NaCl, 0.1% Triton-X-100, 10 mM MgCl₂, 5 mM EDTA) supplemented with 1 mM dithiothreitol (DTT), protease inhibitor cocktail (Sigma, P8340), 1 mM PMSF, 0.1 μM okadaic acid (Calbiochem), 10 mM NaF, 20 mM β-glycerophosphate, and Benzonase (GenScript). Following high-speed centrifugation to remove insoluble debris, the clarified lysates were precleared using either Glutathione Sepharose 4B beads (GE Healthcare) or Amylose Resin (New England Biolabs). The precleared lysates were then incubated for 4 hours at 4°C with beads pre-immobilized with the relevant GST fusion proteins. Subsequently, the beads were washed three times with the lysis buffer, resuspended in standard SDS sample buffer, boiled, and finally analyzed by immunoblotting.

The recombinant plasmids encoding the GST fusion proteins (GST-E2F8-#1, GST-E2F8-#2, GFP-KNTC1-#1, GFP-KNTC1-#2) were commercially designed and synthesized (Fenghbio, Changsha, China). These plasmids were transformed into BL21(DE3) competent cells (TransGen Biotech). Transformed cells were cultured in LB medium with appropriate antibiotics at 37°C until the optical density at 600 nm (OD₆₀₀) reached 0.6-0.8. Protein expression was induced by adding 0.4 mM isopropyl β-D-1-thiogalactopyranoside (IPTG), and the culture was incubated at 16°C for 16 hours. Bacterial cells were collected and lysed via sonication in Buffer A (50 mM Tris-HCl, pH 8.0, 300 mM NaCl, 1 mM EDTA, 1% Triton X-100). The lysate was clarified by centrifugation and then incubated with Glutathione Sepharose 4B beads (GE Healthcare). After extensive washing with the lysis buffer, the bound GST fusion proteins were eluted using a buffer containing 100 mM reduced glutathione.

**Coomassie brilliant blue staining for SDS-PAGE gels**

After SDS-PAGE, the polyacrylamide gel was subjected to Coomassie Brilliant Blue staining for protein visualization. The gel was first fixed and destained in a solution containing 40% methanol and 10% acetic acid for 30 minutes to remove SDS and precipitate proteins. Subsequently, it was incubated with 0.1% Coomassie Brilliant Blue R-250 (ST031, Beyotime) prepared in the same methanol-acetic acid solution for 2 h at room temperature with gentle agitation; for enhanced sensitivity, staining was extended overnight. Following staining, the gel was rinsed briefly with deionized water and then destained in the methanol-acetic acid solution with multiple changes until protein bands became clearly visible against a low-background gel. The destained gel was rinsed again and imaged using a gel documentation system under white light. All solutions were prepared fresh and staining was performed at room temperature with consistent agitation.

**Co-immunoprecipitation coupled with liquid chromatography-tandem mass spectrometry (Co-IP/LC-MS)**

To identify novel protein interactors of KNTC1 in an unbiased manner, co-immunoprecipitation followed by mass spectrometry analysis was performed.

***Cell culture and lysis***: HEK293 or UMUC3 cells were cultured in Eagle's Minimum Essential Medium supplemented with 10% fetal bovine serum (FBS) and 1% penicillin-streptomycin. Cellular propagation was executed under controlled atmospheric conditions (37°C, 5% CO₂, humidity saturation). Cells were screened for *Mycoplasma* contamination biweekly using polymerase chain reaction assays. Cells were harvested and lysed on ice for 30 minutes using a non-denaturing lysis buffer (50 mmol/L Tris HCL pH 8, 150 mmol/L NaCl, 1 mmol/L ethylenediamine tetra-acetic acid (EDTA), 1% NP-40 and 0.25% deoxycholate). The cell lysates were clarified by centrifugation at 14,000 g for 15 min at 4°C. The protein concentration of the supernatant was determined using a BCA assay kit (P0011, Beyotime).

***Co-immunoprecipitation (Co-IP)***: For each immunoprecipitation, 1 mg of total protein lysate was pre-cleared with Protein A/G magnetic beads (P2108-5ml, Beyotime) for 1 h at 4°C. The pre-cleared lysate was then incubated with 2 µg of anti-KNTC1 antibody (Rabbit monoclonal, ab85996, Abcam) conjugated to fresh Protein A/G beads overnight at 4°C with gentle rotation. A critical negative control was set up in parallel using the same amount of normal Rabbit IgG (#2729, Cell Signaling Technology) under identical conditions. After incubation, the beads were washed five times with ice-cold lysis buffer to remove non-specifically bound proteins.

***Sample preparation for MS***: The immunoprecipitated protein complexes on beads were eluted using a low-pH elution buffer or directly denatured in 1× SDS loading buffer. Proteins were reduced with 10 mM DTT, alkylated with 55 mM iodoacetamide, and digested overnight with sequencing-grade trypsin (Promega) at a 1:50 (w/w) enzyme-to-protein ratio. The resulting peptides were desalted using C18 StageTips and vacuum-dried.

***LC-MS/MS analysis***: Peptides were resuspended in 0.1% formic acid and analyzed by nanoflow LC-MS/MS on an Orbitrap Exploris 480 mass spectrometer (Thermo Fisher Scientific) coupled to an EASY-nLC 1200 system. Peptides were separated on a 75 µm × 25 cm, C18 column with a 120 min linear gradient of 5% to 35% acetonitrile in 0.1% formic acid. The mass spectrometer was operated in data-dependent acquisition (DDA) mode. Full MS scans were acquired at a resolution of 120,000 in the Orbitrap, followed by the fragmentation of the top 20 most intense ions via higher-energy collisional dissociation (HCD).

***Database search and identification of specific interactors***: The raw MS data were processed using the MaxQuant software (version 2.0.3.0) and searched against the UniProt Human database concatenated with common contaminants. Trypsin was specified as the protease, allowing up to two missed cleavages. Precursor and fragment ion mass tolerances were set to 10 ppm and 0.02 Da, respectively. Carbamidomethylation of cysteine was set as a fixed modification, and methionine oxidation was set as a variable modification. Protein and peptide spectral match (PSM) identifications were filtered at a false discovery rate (FDR) of 1%.

To distinguish specific KNTC1 binders from background contaminants, the label-free quantification (LFQ) intensity data from the KNTC1-IP sample were rigorously compared with those from the control IgG-IP sample. Candidate interactors were filtered using the following criteria: a. Proteins detected with at least two unique peptides in the KNTC1-IP; b. Significantly enriched in the KNTC1-IP versus the IgG-IP (LFQ intensity ratio > 10 and statistical significance assessed by a Student’s t-test, p < 0.01).

**Synthesis and characterization of PAE@sh-KNTC1 nanoparticles**

Synthesis of PAE was carried out by Michael addition reaction between low molecular weight PEI and PEGDA as reported in our previous paper [3]. Briefly, PEI (2 g, 0.788 mM) and PEGDA (0.788 mM) were separately dissolved in anhydrous dichloromethane (3 mL). The reaction was performed at 45 °C for 48h with shaking. Dichloromethane was removed by vacuum drying for 5 days and the product was dialyzed against distilled water at 4 °C for 24h and lyophilized.

The synthesis of PAE@shRNA was performed according to the previously reported method [4]. For a complex formation study, the shRNA vector solution was added to the PAE (N/P = 45) solution to make a final volume of 10 μL with shRNA concentrations ranging from 100 to 200 nmol and vortex-mixed gently. After 30 min incubation complexes were loaded on 3% agarose gel and subjected to electrophoresis for 30 min at 50 V. The gel was analyzed on UV illuminator after 30 min of EtBr (0.5 mg/mL) soaking.

**Reference**

1. Luo Y, Vlaeminck-Guillem V, Teinturier R, Abou Ziki R, Bertolino P, Le Romancer M, Zhang CX. The scaffold protein menin is essential for activating the MYC locus and MYC-mediated androgen receptor transcription in androgen receptor-dependent prostate cancer cells. Cancer Commun (Lond). 2021 Dec;41(12):1427-1430. doi: 10.1002/cac2.12217. Epub 2021 Dec 1. PMID: 34850609; PMCID: PMC8696212.
2. Malbeteau L, Poulard C, Languilaire C, Mikaelian I, Flamant F, Le Romancer M, Corbo L. PRMT1 Is Critical for the Transcriptional Activity and the Stability of the Progesterone Receptor. iScience. 2020 Jun 26;23(6):101236. doi: 10.1016/j.isci.2020.101236. Epub 2020 Jun 4. PMID: 32563156; PMCID: PMC7305383.
3. Park MR, Han KO, Han IK, Cho MH, Nah JW, Choi YJ, Cho CS. Degradable polyethylenimine-alt-poly(ethylene glycol) copolymers as novel gene carriers. J Control Release. 2005 Jul 20;105(3):367-80. doi: 10.1016/j.jconrel.2005.04.008. PMID: 15936108.
4. Jere D, Xu CX, Arote R, Yun CH, Cho MH, Cho CS. Poly(beta-amino ester) as a carrier for si/shRNA delivery in lung cancer cells. Biomaterials. 2008 Jun;29(16):2535-47. doi: 10.1016/j.biomaterials.2008.02.018. Epub 2008 Mar 3. PMID: 18316120.
